# Molecular insights into juvenile hormone maturation by juvenile hormone acid methyltransferase

**DOI:** 10.1016/j.jbc.2026.111474

**Published:** 2026-04-17

**Authors:** Marie-Ève Picard, Michel Cusson, Rong Shi

**Affiliations:** 1 Département de biochimie, de microbiologie et de bio-informatique, Institut de Biologie Intégrative et des Systèmes (IBIS), Université Laval, Quebec City, Quebec, Canada; 2 PROTEO-Regroupement Québécois de Recherche sur la Fonction, l’Ingénierie et les Applications des Protéines, Université Laval, Quebec city, QC, Canada; 3 Natural Resources Canada, Canadian Forest Service, Laurentian Forestry Centre, Quebec City, Quebec, Canada

**Keywords:** juvenile hormone (JH), insect, protein structure, S-adenosyl-L-methionine (SAM), X-ray crystallography

## Abstract

Juvenile hormone acid methyltransferase (JHAMT) is an enzyme involved in the biosynthesis of juvenile hormone (JH) in insects, catalyzing the methylation of farnesoic acid and JH acids to produce active JHs. Given its important role in JH biosynthesis, JHAMT has attracted significant interest as a potential target for pest control strategies. Inhibiting JHAMT activity could disrupt normal JH production, leading to developmental abnormalities and reduced reproductive success in pest species. We have determined the crystal structure of a JHAMT from the spruce budworm *Choristoneura fumiferana* (CfJHAMT) in complex with the cofactor product S-adenosyl-L-homocysteine (SAH) and the substrate juvenile hormone acid III, at a resolution of 1.77 Å, and in the presence of SAH alone. Structural and biochemical analyses, supported by site-directed mutagenesis, revealed key residues involved in cofactor and substrate recognition. A proximity-based catalytic mechanism is proposed wherein critical interactions position the substrate and cofactor for methyl group transfer. These findings contribute to our understanding of the structure-function relationship of CfJHAMT and offer preliminary structural insights that may assist in the development of inhibitors, which could potentially be used to target JH biosynthesis in pest insects.

Juvenile hormones (JHs) are a group of acyclic sesquiterpenoids that play a crucial role in the regulation of development, reproduction, and other physiological processes in insects. These hormones are key regulators in maintaining the larval state, coordinating molting, and controlling metamorphosis (1, 2).

The biosynthesis of juvenile hormones occurs in the corpora allata (CA), a pair of endocrine glands associated with the insect brain. The insect-specific portion of the biosynthetic pathway involves a series of enzymatic reactions that convert farnesyl diphosphate (FPP) into JHs. Among the enzymes involved, juvenile hormone acid methyltransferase (JHAMT) catalyzes the methylation of farnesoic acid (FA) and juvenile hormone acids (JHAs) into their active methyl ester forms (Fig. 1), essential for the production of bioactive JH, which can then exert its physiological effects on the insect (1).

**Figure 1:**
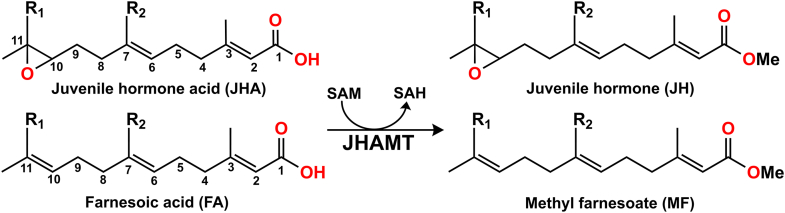
**JHAMT catalysis on juvenoid acids (JH acids and FAs).** Substrate nomenclature by R_1_ and R_2_: R_1_ = R_2_ = H, JHA III and FA; R_1_ = CH_3_, R_2_ = H, JHA II and homo-FA; R_1_ = R_2_ = CH_3_, JHA I and bishomo-FA.

Biochemical characterization and functional analyses of JHAMT have been conducted on various insect species, revealing its conserved yet taxon-specific roles in developmental transitions and physiological adaptations. A notable example is the silkworm *Bombyx mori*, where the JHAMT gene was first identified in the CA. In this insect, the expression of BmJHAMT and its enzymatic activity are closely linked to developmental stages. The recombinant BmJHAMT protein, expressed in *Escherichia coli*, efficiently converts FA and JHA I–III into their respective methyl esters, confirming the enzyme’s role in JH activation (3). Expression patterns of the BmJHAMT gene show high activity during the third and fourth larval instars, followed by a sharp decline in the final instar, aligning with the cessation of JH biosynthesis and the initiation of metamorphosis. This temporal regulation underscores the importance of JHAMT suppression to enable the transition from larva to pupa, a finding consistent with decreased JH biosynthetic activity during this phase (4).

Studies in other insect models corroborated the centrality of JHAMT in JH biosynthesis. For instance, *Drosophila melanogaster* JHAMT (DmJHAMT) has been shown to be expressed predominantly in the ring gland (containing the corpora allata), with its expression pattern reflecting JH levels throughout development. Overexpression of DmJHAMT results in lethal phenotypes, similar to those induced by JH analogs, insect growth regulators used in pest control, while the outcome of RNA interference (RNAi) of DmJHAMT points to possible compensatory pathways that mitigate its loss (5). Similarly, in the red flour beetle *Tribolium castaneum*, RNAi targeting the JHAMT ortholog TcMT3 leads to precocious metamorphosis, further confirming the enzyme’s role in maintaining the larval stage (6).

Recombinant BmJHAMT exhibited higher conversion efficiency for ethyl-substituted JHA I (C-7, C-11) and JHA II (C-11) than with JHA III and FA and appears to play a rate-limiting role in *B. mori* (3). The recombinant proteins of orthologues of JHAMT from *T. castaneum* (6), *D. melanogaster* (5), and the yellow fever mosquito *Aedes aegypti* (7) can methylate JHA III and FA with comparable efficiency. In *A. aegypti*, JHAMT (AaJHAMT) exhibits higher affinity for FA but a faster turnover rate for JHA III (8). However, in this species, JHAMT transcript levels did not consistently correlate with *in vitro* JH biosynthesis, indicating that it may not function as the rate-limiting enzyme in this species (7). In the corpora allata of different insect species, the order of the last two steps of JH biosynthesis also shows an apparent difference: in Lepidoptera, epoxidation of FA to JHA is generally considered to occur before methylation, whereas in several other groups (*e.g.*, Orthoptera, Dictyoptera, Coleoptera, and Diptera) methylation is generally considered to precede epoxidation (1, 9).

The functional significance of JHAMT extends beyond development to reproductive regulation and environmental responsiveness. In the German cockroach, *Blattella germanica*, JHAMT activity integrates nutritional signals mediated through the insulin receptor and TOR pathways, functioning as a hormonal checkpoint that links nutritional status to JH production (10). Similar connections between JHAMT expression and reproductive cycles have been observed in *Diploptera punctata* (11). JHAMT’s higher expression in queen larvae compared to worker larvae, in the honey bee *Apis mellifera*, suggests it plays a crucial role in honeybee caste differentiation (12).

Given its central role in JH biosynthesis, JHAMT has attracted significant interest as a potential target for the development of pest control products. Inhibiting JHAMT activity could disrupt normal JH production, leading to developmental abnormalities and reduced reproductive success in pest species (13). Thus, detailed studies on JHAMT structure, function, and regulation are crucial for developing novel insect management approaches.

In recent research, Guo *et al.* (14) provided a comprehensive analysis of juvenile hormone acid methyltransferases in *B. mori* (BmJHAMT1, referred to above as BmJHAMT, and BmJHAMT2) by revealing the three-dimensional structures of the BmJHAMT2 apoenzyme, the holoprotein in a binary complex with its cofactor product S-adenosyl-L-homocysteine (SAH), and the ternary complex comprising SAH and its product methyl farnesoate (MF).

In this work, we aim to expand knowledge on insect JHAMT through a comprehensive structural and functional analysis of *Choristoneura fumiferana* JHAMT (CfJHAMT). Commonly known as the spruce budworm, *C. fumiferana* is a destructive forest pest responsible for widespread defoliation of coniferous trees in North America (15). Here, we present the crystal structures of CfJHAMT in complex with its co-product, S-adenosyl-L-homocysteine, and substrate, JHA III, and in binary complexes with SAH. This structural analysis is complemented by site-directed mutagenesis to identify critical residues driving catalysis, suggesting a proximity-based mechanism. By elucidating the molecular details of JHAMT, including its functional domains and putative catalytic mechanism, we provide insights into its role in juvenile hormone biosynthesis and its potential as a target for the design of novel insecticidal molecules.

## Results

### Identification and functional characterization of JHAMT in *C. fumiferana*

To identify the putative JHAMT of the spruce budworm, the transcriptome was analyzed, revealing three contigs encoding homologs of lepidopteran JHAMTs. Although these three paralogs exhibited relatively high similarity to one another, they displayed limited similarity to the well-characterized JHAMT from *B. mori* (3), which is exclusively expressed in the CA and is involved in JH biosynthesis. Given the potential rarity of the "true" CfJHAMT transcript, likely due to the limited representation of CA tissue in the whole insect transcriptome, a search of the spruce budworm genome assembly (16) was conducted for a homolog more closely related to BmJHAMT1. This search identified a genomic CDS as the most likely candidate for CfJHAMT. Notably, this candidate is the only paralog containing a glutamine residue at position 14 (Gln14; Cf numbering), which has been shown to be essential for catalysis in *B. mori* JHAMT1 (14). It remains unclear whether the three transcriptome-derived paralogs and the genomic candidate have distinct or overlapping functions, though the conserved catalytic residue in the latter suggests it is the most likely catalytically active JHAMT.

To evaluate the functionality of this putative CfJHAMT, its ability to methylate juvenoid acids was tested *in vitro*. A recombinant N-terminal hexahistidine-tagged protein was synthesized by cloning the coding sequence into the expression vector pET28a and expressing it in *E. coli*. The enzyme's activity was assessed using reversed-phase high-performance liquid chromatography (RP-HPLC) with JHA III and FA as substrates and S-adenosyl-L-methionine (SAM) as the cofactor. Representative chromatograms of substrate controls, authentic product standards (JH III and MF), and enzyme reactions are shown in the Supplementary Information (Figs. S1 and S2). Major peaks with retention times matching the standards confirmed the methylation of JHA III (Fig. S1) and FA (Fig. S2), indicating that CfJHAMT functions as a SAM-dependent methyltransferase capable of catalyzing juvenoid acid methylation.

### Structural overview and comparison of binary and ternary complexes of CfJHAMT

Here, we present the first JHAMT substrate-bound crystal structure, in a ternary complex comprising CfJHAMT, SAH, and JHA III (CfJHAMT-SAH-JHA III; PDB 9XYS). We also determined two crystal structures of the CfJHAMT-SAH binary complex: one used for initial phasing despite significant disorder (crystal form 1; PDB 9XYO; Fig. S3), and one named CfJHAMT-SAH (crystal form 2; 9XYQ). All structures have one molecule in the asymmetric unit, except CfJHAMT-SAH for which four molecules are found. In every instance, CfJHAMT appears to be a monomer, which is consistent with our results from size-exclusion chromatography (Fig. S4). In contrast, the closely related BmJHAMT2, with which CfJHAMT displays 38% sequence identity, was reported to form a homodimer.

**Figure 2:**
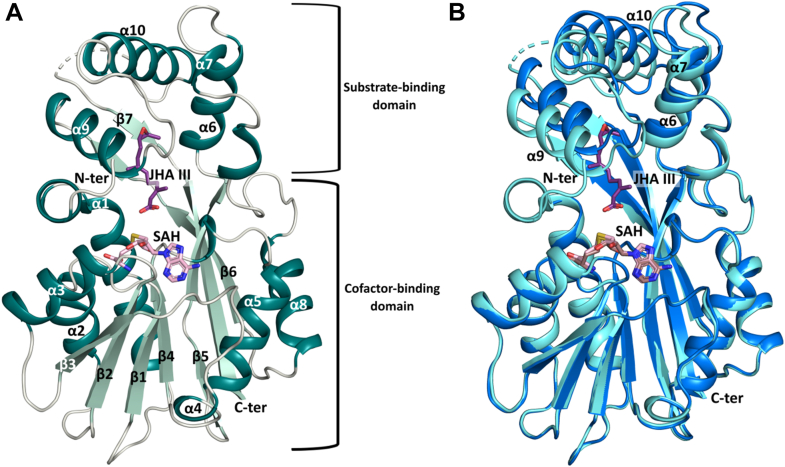
**Global structure of CfJHAMT.***A*, Cartoon representation of CfJHAMT colored by secondary structure. Cofactor product SAH and substrate JHA III molecules are shown in stick mode. The nomenclature of each helix and beta strand is indicated (α1 to α10; β1 to β7). The disorder segment ^249^FTNNNTKE^256^ between helix α10 and beta strand β7 is represented by the dotted curve. *B*, structural comparison between CfJHAMT bound to SAH alone and to both SAH and substrate JHA III. The binary CfJHAMT–SAH structure is shown in *dark blue* (cartoon representation), while the ternary CfJHAMT–SAH–JHA III complex is shown in *light blue*. Binding of JHA III induces a transition into a more closed conformation, characterized by the inward movement of helices α9 and α10, and, to a lesser extent, α6.

CfJHAMT-SAH showed density beginning at residue 11, with a missing segment from Phe249 to Val260. In the CfJHAMT-SAH-JHA III crystal structure, density was present starting at residue 1, though a gap was observed for residues Phe249 to Glu256 (Fig. 2*A*). Structural superposition of both structures reveals an r.m.s.d. of 0.83 Å calculated for the Cα atoms over 248 residues, reflecting structural adjustments from cofactor to substrate binding, a point that is dealt with in more detail below (Fig. 2*B*).

### CfJHAMT is a class I SAM-dependent MTase

Consistent with the structural features of this class of methyltransferases (MTases), CfJHAMT exhibits a characteristic Rossmann-like fold architecture (17), classifying it as a Class I SAM-dependent MTase. This fold features a central seven-stranded β-sheet flanked by surrounding α-helices, forming the distinctive open αβ sandwich topology that provides the cofactor-binding domain (Fig. 2*A*). Here, the α-helices are arranged with α1–α3 on one side and α4, α5, and α8 on the opposite side of the β-sheet.

An additional domain, referred to as the substrate-binding domain, extends above the Rossmann-like core region, incorporating two distinct insertions that, as observed in other MTases, contribute to substrate selectivity (17). The first insertion comprises α6 and α7 (His144 to Asp176), located between β5 and α8. The second insertion consists of α9, α10, a highly flexible region (Ser204 to Ser263), situated between β6 and β7. These insertions were previously described by Guo *et al.* (14) as functioning like a "cap" over the cofactor-binding domain. Together, the Rossmann-like domain and the substrate-binding domain are separated by a broad cleft that serves as the binding site for both the cofactor and the substrate (Fig. 2*A*).

### Significant domain movement upon substrate binding in CfJHAMT

The conformational states of the CfJHAMT-SAH and CfJHAMT-SAH-JHA III protein structures were analyzed to determine the influence of substrate binding on structural dynamics. In the presence of SAH and the absence of JHA III, the cap formed by the substrate-binding domain can be classified as "open", while a "closed" conformation emerged in the presence of JHA III (Fig. 2*B*). The dynamics of the N-terminal segment (Met1-Asn9) appear to remain unaffected by the presence of SAH alone. Upon JHA III binding, the structure transitions to a more rigid "closed" conformation, likely favoring reactivity with SAM. These findings underscore the critical role of substrate in modulating conformational states and suggest that structural flexibility facilitates functional interactions in the closed conformation.

### Key interactions in the SAH coproduct binding pocket of CfJHAMT

To investigate cofactor binding and its role in juvenoid acid methylation by CfJHAMT, we superimposed the CfJHAMT-SAH and CfJHAMT-SAH–JHA III structures and analyzed protein–cofactor interactions. Both complexes exhibited nearly identical interaction patterns with the protein. The CfJHAMT-SAH-JHA III crystal was prepared through cocrystallization with SAH and JH III, followed by soaking with JHA III, as similar crystal setups did not exhibit any density for JH III.

**Figure 3:**
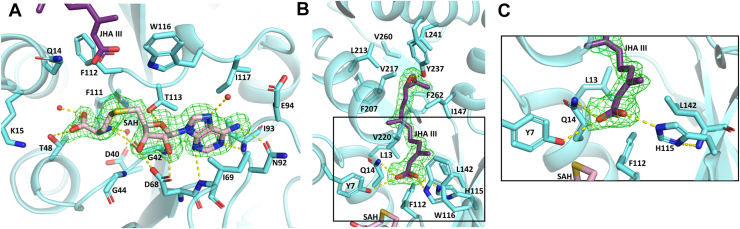
**Structural features of the SAM and JHA III binding pockets in CfJHAMT.** (*A*) SAM and (*B*) JHA III binding sites in CfJHAMT. The cofactor product SAH (*pink*) and substrate JHA III (*purple*) are depicted in *stick* representation. Water molecules interacting with SAH are shown as *red spheres*, and hydrogen bonds are indicated by *yellow dashed lines*. The Fourier difference map (Fo − Fc) with SAH or JHA III omitted from refinement is colored in *green* and contoured at 2.5σ. *C*, close-up of CfJHAMT interactions with the carboxylic acid moiety of JHA III. Trp116 is omitted for clarity.

The hallmark of the SAM binding domain in Class I SAM-dependent MTase is the presence of conserved structural motifs, notably the loosely conserved glycine-rich GxGxG (G42-X-G44-X-G46; Cf numbering) sequence (17) within the first β-sheet and a critical acidic residue, Asp68 in CfJHAMT, located at the terminus of the β2 strand. Asp68 forms hydrogen bonds with the 2′- and 3′-hydroxyl groups on the ribose ring of the SAH molecule, while the ring oxygen hydrogen-bonds with Thr113 (Fig. 3*A*). Additionally, residues such as Asp40 in Motif I play a pivotal role by polarizing water molecules to facilitate coordination with the free nitrogen of the SAH’s homocysteine moiety (17, 18). The carboxyl group of SAH interacts with Thr48 and a water molecule, while the amino group of the methionine moiety forms hydrogen bonds with the main chain carbonyls of Gly42 and Phe111, as well as with adjacent water molecules. The adenine base is engaged in multiple hydrogen bonds: the pyrimidine nitrogen atom N1 interacts with the main chain nitrogen of Ile93, while N3 forms a weak hydrogen bond with the nitrogen of Ile69. The exocyclic N6 amino group interacts with the side chain oxygen of Asn92. In the ternary complex CfJHAMT-SAH-JHA III structure, an additional hydrogen bond is observed between the nitrogen N7 of the adenosine base’s imidazole ring and a nearby water molecule. The adenine ring is positioned in a hydrophobic cavity formed by Thr113, Trp116, Ile117, Glu94, Asn92, Ile93, Ile69, and Asp68, while the homocysteine moiety is surrounded by Phe112, Gln14, Lys15, Thr48, and Gly44 (Fig. 3*A*). All these interactions allow for the sulfur atom from the SAH to be positioned in proximity of the JH binding site.

### Structural basis of substrate JHA III stabilization

The binding of JHA III in the substrate-binding domain that extends above the Rossmann-like core region is characterized by polar interactions with the acid function and hydrophobic interactions with the terpenoid skeleton. The carboxylic acid moiety, as well as the terpenoid backbone, has well-defined electron density and exhibits a conformation similar to that observed for JH III when bound to the mosquito JH-binding protein (PDB Code: 5V13) (19). The carboxylic acid group is stabilized in proximity to the sulfur group of the cofactor SAH through direct hydrogen bonds involving the oxygen atoms of the carboxylic acid group and the Nε2 atom of His115, the Nε1 atom of Trp116, the hydroxyl oxygen of Tyr7, and the amide nitrogen of Gln14, as well as additional stabilization provided by van der Waals interactions with those same nearby residues (Fig. 3, *B* and *C*). The distance between the carboxylate group and the sulfur atom of SAH is approximately 4.6 Å, which is consistent with geometries observed in other SAM-dependent methyltransferases (17). The terpenoid core of the substrate is positioned in a hydrophobic pocket formed by residues Phe112, Ile147, Val217, and Val220 on one side, and Leu13, Leu142 and Phe262 on the other. Furthermore, the methyl group adjacent to the epoxide is stabilized by hydrophobic contacts with Phe207, Leu213, Leu241, Val260, and Phe262. The epoxide group forms a hydrogen bond with the phenolic hydroxyl of Tyr237, contributing to ligand anchoring. The JHA III molecule present in the structure exhibits a 10R configuration (Fig. 3*B*). This stereochemistry is consistent with findings from studies in other species, which demonstrate moderate to high stereospecificity favoring the 10R configuration (5, 6, 7).

**Figure 4:**
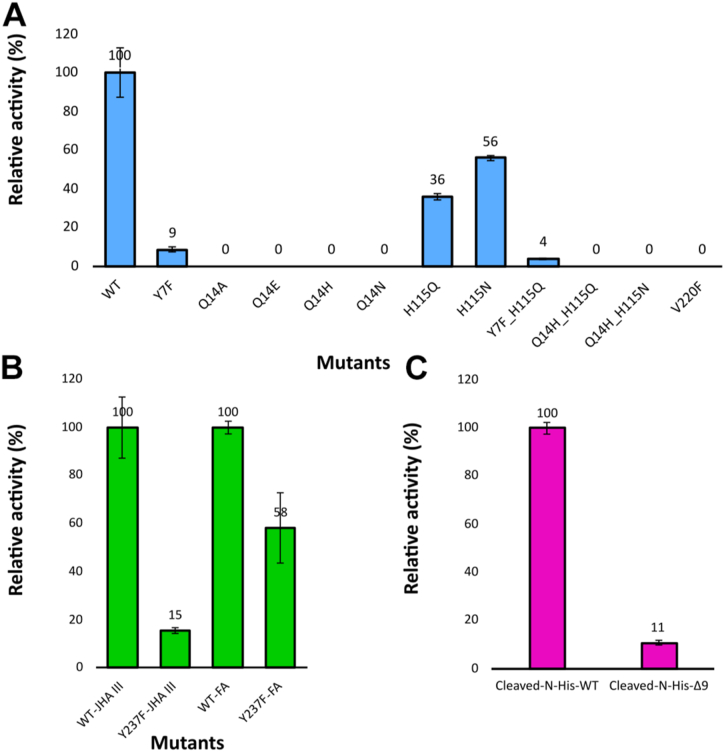
**Comparative analysis of relative activity for wild-type CfJHAMT and selected mutants.***A*, relative activity of mutant proteins expressed as the percentage of JH III production by mutant CfJHAMT compared to wild-type CfJHAMT (WT) under identical experimental conditions. *B*, relative activity of the Y237F, expressed as the percentage of JH III and MF production compared to WT. *C*, effects of N-terminal truncation on CfJHAMT activity with JHA III as substrate, comparing cleaved His-tag CfJHAMT (Cleaved-N-His-WT) and a nine-residue N-terminal deletion (Δ9-Cleaved-N-His). Assays were conducted using the standard CfJHAMT protocol in 50 mM HEPES buffer (pH 8.0) containing 3 μM enzyme (WT or mutant), 400 μM SAM, and 200 μM JHA III substrate, incubated at 25 °C for 5 min. Control reactions excluded the enzyme. Each experiment was performed in triplicate. Mean and standard deviations are shown.

To investigate the functional significance of specific amino acid residues involved in substrate binding, mutational analyses were conducted, accompanied by relative *in vitro* activity assays using JHA III as the substrate, with wild-type activity normalized to 100% (Fig. 4*A*).

Among the residues investigated, the invariant residue among homologous sequences Gln14 is shown to be critical for activity. The Q14E mutation, which replaces glutamine with glutamate, disrupts the hydrogen bond and completely abolishes enzymatic activity, highlighting the necessity of this interaction for catalysis. Similarly, the Q14N mutation results in a complete loss of methylation activity, which is likely due to the shorter side chain of asparagine being unable to form a stable hydrogen bond with JHA III. Furthermore, the Q14H mutation also inactivates the enzyme, possibly because the bulkier side chain of histidine introduces steric hindrance that interferes with proper substrate positioning. The terpenoid backbone is positioned within a tunnel formed by Leu142 on one side and Val220 on the other (Fig. 3*B*). Substitution of Val220 with the bulkier residue Phe obstructs the channel, imposing significant steric constraints that hinder substrate access or positioning. As a result, the enzyme is rendered inactive.

His115 is positioned within hydrogen-bonding distance of the carboxylic acid group of JHA III, the site of methylation by JHAMT (Fig. 3, *B* and *C*). The H115Q mutation, which substitutes histidine with glutamine, retains 36% of wild-type activity, suggesting that changes in hydrogen-bonding properties or steric effects partially disrupt substrate interaction. In contrast, the H115N mutation, replacing histidine with asparagine, retains 56% of wild-type activity, indicating that asparagine partially preserves critical interactions required for activity. The design of H115N and H115Q was guided by the residues naturally present at this position in homologous sequences (see sequence alignment, Fig. S5).

### Tyr237 anchors the epoxide moiety of substrate JHA III

The hydrophobic tail of JHA III features an epoxide group that interacts with Tyr237, as previously described. To explore the catalytic importance of Tyr237, this residue was mutated to Phe to retain aromatic properties while removing the hydroxyl group, thereby eliminating its hydrogen-bonding capability. Enzymatic activity assays were conducted to evaluate relative activity. For the wild-type enzyme, methylation of JHA III was set as the reference at 100% relative activity. The Y237F mutant exhibited a marked reduction, retaining only 15% relative activity with JHA III, highlighting Tyr237's critical role in substrate binding and catalysis (Fig. 4*B*). Activity was also assessed using the non-epoxide-containing substrate farnesoic acid (FA), with wild-type activity similarly normalized to 100%. The Y237F mutant showed a less severe activity reduction, retaining 58% relative activity with FA (Fig. 4*B*). These results underscore the significance of Tyr237's hydroxyl group in facilitating effective interaction with the epoxide moiety of JHA III. In contrast, the moderate reduction in activity with FA suggests that the absence of an epoxide group alters the binding interaction, rendering Tyr237 less essential for catalysis in this context.

### Impact of N-terminal deletion and mutation on enzymatic function

Structural analysis of CfJHAMT revealed that the N-terminal region (Met1-Asn9) exhibits complete density in the ternary complex CfJHAMT-SAH-JHA III. However, the density for the first 11 residues is missing in the binary structures, suggesting potential structural flexibility or instability in the absence of the substrate. To investigate the functional role of the N-terminal region, a construct with the deletion of the first nine residues (CfJHAMT_Δ9) was created. This construct retained the residues “GSH” from the pET28a vector following thrombin cleavage of the His-tag, ensuring the authentic CfJHAMT sequence began immediately after.

Comparative enzymatic assays between cleaved wild-type CfJHAMT and cleaved CfJHAMT_Δ9 demonstrated a significant loss of activity for the Δ9 construction, which retained only 11% of the relative activity (Fig. 4*C*). These results highlight the critical importance of the N-terminal residues in enzymatic function.

In the context of identifying which specific contribution within the N-terminal region is important, structural analysis of the CfJHAMT-SAH-JHA III structure showed that Tyr7 directly interacts with the reactive carboxylic acid group of JHA III *via* hydrogen bonding, an interaction deemed important for enzymatic activity. Substitution of Tyr7 with Phe, which only removes the hydroxyl group required for hydrogen bond formation, resulted in a severe reduction in activity, retaining only 9% of the wild-type enzymatic activity (Fig. 4*A*). These findings underscore the significant role of the N-terminal region, particularly Tyr7, in maintaining the catalytic function of CfJHAMT, as reflected in both structural and enzymatic assays.

### Structural comparison of CfJHAMT and BmJHAMT2

Structural comparison of CfJHAMT with other proteins using the DALI server identified its closest structural homolog as being BmJHAMT3 (inactive paralog of BmJHAMT1 and 2, PDB 7V2S, r.m.s.d.: 1.7 Å: Z-Score: 33.9) from *B. mori*. The next three structural homologs are methyltransferases, including BmJHAMT2 (PDB 7EBS; r.m.s.d.: 2.0 Å; Z-score: 29.7), with an r.m.s.d. ranging from 2.0 to 3.6 Å for the corresponding Cα atoms. Notably, all these enzymes share a nearly identical Rossmann-fold architecture in their cofactor-binding domains (Fig. S6). As anticipated, given their differing substrate specificities, the most pronounced structural variations among these proteins are localized within the substrate-binding domain.

**Figure 5:**
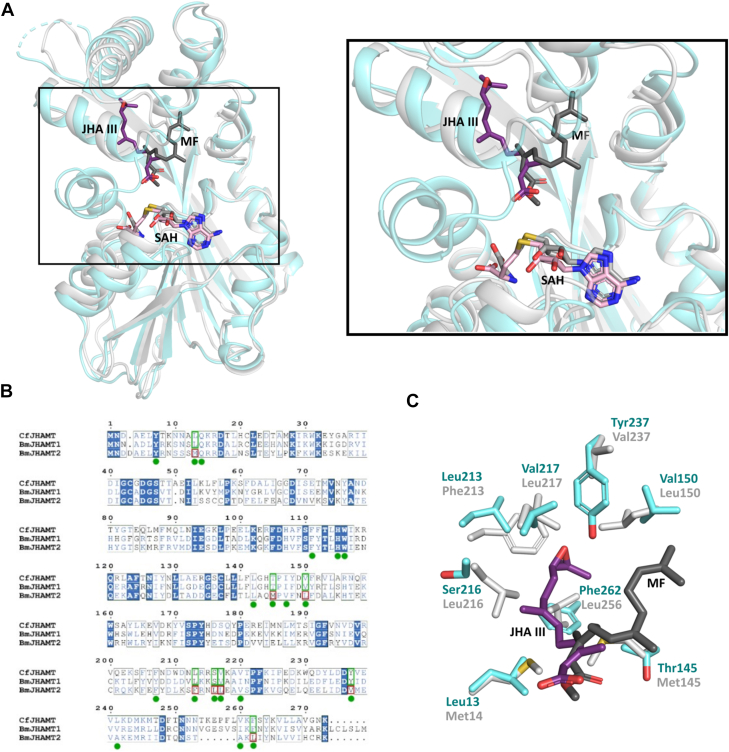
**Comparative analysis of CfJHAMT, BmJHAMT1 and BmJHAMT2.***A*, global structural alignment of CfJHAMT (*light blue*) and BmJHAMT2 (*gray*), shown as cartoons. SAH is depicted as *pink sticks*, with JHA III (CfJHAMT) and MF (BmJHAMT2) represented as *purple* and *dark gray sticks*, respectively. Close-up views of the JHA III tunnel in CfJHAMT and the FA tunnel in BmJHAMT2. *B*, sequence alignment of CfJHAMT, BmJHAMT1, and BmJHAMT2. Residues forming the JHA III binding site are marked with *green circles*. Positions conserved between CfJHAMT and BmJHAMT1 but differing in BmJHAMT2 are highlighted with *green* and *red* rectangles, respectively. *C*, structural superposition of the CfJHAMT ternary complex (*light blue*) and BmJHAMT2 (*gray*; PDB: 7EC0_chainG), highlighting residue differences in BmJHAMT2 within the JHA III binding site.

From a global alignment between CfJHAMT and BmJHAMT2 (PDB 7EC0_chainG), we can see that the Rossmann-like domain, which hosts the SAM-binding site, is well aligned in both structures. The top region, including the two insertions into the Rossmann-like domain that form the substrate-binding pocket, is also overall well conserved. A closer inspection highlights two distinct binding configurations, suggesting the presence of two different binding sites: one for JHA III in CfJHAMT and one for MF in BmJHAMT2 (Fig. 5*A*).

Further analysis of these two binding sites revealed that BmJHAMT2 lacks several residues identified as important for JHA III binding. Notably, BmJHAMT2 often has larger side chains at these positions, thereby reducing the available space for JHA III. Specifically, Thr145, Val150, Leu213, Ser216, Val217, and Phe262 in CfJHAMT are replaced by Met145, Leu150, Phe213, Leu216, Leu217, and Leu256 in BmJHAMT2, respectively (Fig. 5, *B* and *C*). These substitutions restrict the JHA III pocket in BmJHAMT2, suggesting that significant conformational adjustments would be required for BmJHAMT2 to accommodate JHA III. Most importantly, the critical Tyr237, which is important for JHA III binding in CfJHAMT and other JHBPs, is replaced by Val237 in BmJHAMT2. Of particular interest, all residues that diverge between CfJHAMT and BmJHAMT2 in the JHIII acid-binding site are conserved between CfJHAMT and BmJHAMT1. This supports the notion that BmJHAMT1’s structure is likely more similar to CfJHAMT than BmJHAMT2, consistent with its known activity of methylating JH acids.

### A putative proximity-driven catalytic mechanism in CfJHAMT

**Figure 6:**
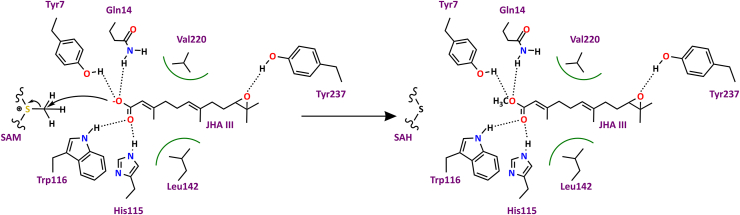
**Proposed catalytic mechanism of CfJHAMT.** Key residues Gln14, His115, and Tyr7 are shown stabilizing the substrate through hydrogen bonding, facilitating substrate alignment and methyl transfer. The methylation reaction involves an SN2 nucleophilic attack on the methyl group of SAM, leading to C-S bond cleavage and methyl group transfer. Leu142 and Val220 stabilize the terpenoid backbone, while Tyr237 interacts with and stabilizes the epoxide group of the substrate.

The ternary structure of CfJHAMT, with the cofactor product SAH and substrate JHA III bound, along with the mutational analysis, serves as a valuable model for investigating the molecular mechanisms of substrate recognition and enzymatic catalysis (Fig. 6).

Gln14 plays a pivotal role in the catalytic mechanism of CfJHAMT, as evidenced by the mutational analyses, and forms a hydrogen bond with the negatively charged oxygen (O^-^) of JHA III. At the assay pH of 8.0, the carboxylic acid group of JHA III is expected to be deprotonated, forming a negatively charged carboxylate (COO^-^) group, consistent with typical carboxylic acid pKa values. The interaction between Gln14 and the carboxylate group likely stabilizes the transition state during the reaction, facilitating the transfer of a methyl group from the cofactor S-adenosylmethionine (SAM) to the substrate.

Natural product methyltransferases are known to catalyze transmethylation reactions through three recognized mechanisms: (i) a proximity and desolvation mechanism, where the substrate reacts with a methyl donor such as SAM upon proper alignment, (ii) a base-mediated mechanism, in which a base abstracts a proton from the substrate to facilitate its reaction, and (iii) a metal-dependent mechanism involving catalytic roles of metal ions (20). Regarding CfJHAMT, the metal-dependent mechanism is unlikely, as metal chelator EDTA (100x protein concentration) does not affect activity (Fig. S7), and no metals are observed in the active site region of available JHAMT structures. This points to a reliance on proximity/desolvation or base-mediated catalysis.

Within the binding site, His115 is strategically positioned near the acid moiety of JH and was previously proposed to act as a conjugate base, activating the substrate *via* proton abstraction (14). However, our structural and mutational analyses suggest a different role for His115 in CfJHAMT. Structural analysis reveals that His115 forms a hydrogen bond between its proximal nitrogen Nδ1 and the backbone NH of Leu142, with a measured distance of 3.1 Å, consistent with a hydrogen-bond-accepting interaction (Fig. 3*C*). Given histidine's chemical properties and pKa behavior, its distal nitrogen (Nε2) is the most likely site to bear the proton. This distal nitrogen is positioned 2.7 Å from the acid moiety of JHA III, a distance indicative of a strong hydrogen bond. This interaction suggests that the acid moiety, likely in its deprotonated (negatively charged) form, is stabilized by His115 and primed for direct reaction with the methyl group from SAM, most consistent with a proximity-driven mechanism.

The partial retention of activity in the H115N mutant indicates that His115’s role is primarily to ensure effective hydrogen bonding and spatial organization, rather than relying on histidine’s unique conjugated base chemical properties. However, the ∼64% reduction in activity in the H115Q mutant underscores the important nature of these interactions. Thus, while His115 itself is not strictly irreplaceable, this residue helps orient the substrate and facilitates the catalytic process.

The double mutants Q14H/H115N and Q14H/H115Q exhibit no enzymatic activity, further highlighting the critical roles of Gln14 and His115 in catalysis. In the Q14H/H115Q mutant, where the amino acids were effectively exchanged, the complete loss of activity suggests that the precise spatial arrangement of these residues, rather than their individual chemical properties, is primordial for catalysis. The double mutant Y7F_H115Q retains 4% of its catalytic activity, indicating that Tyr7 is less critical to the mechanism than Gln14.

In summary, the proposed mechanism of CfJHAMT aligns with the principles of the proximity and desolvation mechanism. This putative mechanism does not involve the direct participation of catalytic groups within the enzyme. Instead, the enzyme's active site architecture and chemical environment position the acceptor (JHA III) near the methyl donor (SAM) in an orientation optimized for nucleophilic substitution. The reaction proceeds through an SN2 nucleophilic attack on the methyl group of SAM, resulting in the cleavage of the C-S bond and transfer of the methyl group to JHA III. Furthermore, the absence of water from the donor-acceptor interface (desolvation) enhances catalytic efficiency (20). Critical residues like Gln14 and His115, aided by Tyr7, contribute by stabilizing the substrate through hydrogen bonding, ensuring precise spatial alignment, and creating an optimal environment for this methyl transfer (Fig. 6).

## Discussion

This study reports on the identification and comprehensive characterization of CfJHAMT, a SAM-dependent methyltransferase from the spruce budworm, *C. fumiferana.* CfJHAMT catalyzes the methylation of juvenoid acids in the biosynthetic pathway of JH biosynthesis. In this work, we present the structural elucidation of CfJHAMT in the presence of SAH alone and in the presence of SAH and substrate JHA III. This enabled a detailed experimental visualization of the binding site architecture, specifically revealing the location of the substrate JHA III, within the substrate-binding pocket and offering insights into the enzyme’s catalytic mechanism.

We present the first substrate-bound structure of CfJHAMT in complex with coproduct SAH and substrate JHA III. This structure reveals the structural rigidity of the SAM-binding site within a Rossmann-like alpha-beta fold. Conversely, the substrate-binding domain exhibits somewhat more flexibility, which appears to be crucial for accommodating the substrate and stabilizing the substrate-binding domain upon substrate binding. Notably, the analysis supports the existence of distinct "open cap" and "closed cap" conformational states.

Structural and biochemical studies confirmed CfJHAMT's enzymatic activity and its structure-activity relationships, highlighting the role of strictly conserved residues Gln14 and His115 (Fig. S8). Mutational studies of Gln14 (substituted with A/E/H/N) consistently resulted in a complete loss of enzymatic activity, emphasizing its essential role in stabilizing the negatively charged carboxylate group of JHA III. Similarly, His115 was investigated through mutational analysis and is hypothesized to facilitate proper hydrogen bonding and spatial alignment of the substrate. Notably, the H115N mutant retained substantial activity, likely indicating that His115 primarily contributes structurally rather than functioning as a proton-transferring residue. A comprehensive sequence alignment of over 500 homologous JHAMT sequences retrieved from the non-redundant protein database revealed that the histidine residue at position 115 in CfJHAMT is not strictly conserved across insects (Fig. S6). In fact, multiple species possess a glutamine or asparagine at the equivalent position. This variation aligns well with our proposed mechanism, wherein His115 primarily contributes to substrate stabilization through hydrogen bonding. Residues such as Gln or Asn, which retain the capacity for hydrogen-bonding interactions, could similarly fulfill this stabilizing role. Consequently, the observed sequence divergence further supports the notion that the essential feature of this position is not the histidine side chain *per se*, but rather the ability to provide a polar contact that ensures precise substrate positioning for effective methyl transfer. Consistently, in the canonical JHAMT of *T*. *castaneum* JHAMT, substitution of the corresponding His with alanine (H114A) completely abolished methylation activity, whereas replacement with asparagine (H114N) preserved enzymatic function (21). Similar to our CfJHAMT, optimal orientation of the substrate carboxylate moiety by a His residue has been previously observed in loganic acid O-methyltransferase (PDB 6C8R), and His162 in this enzyme does not serve as general base (22, 23). Moreover, the “proximity and desolvation” mechanism has also been proposed for other carboxylic acid O-methyltransferases, such as the mycobacterial fatty acid O-methyltransferase (MmFAMT, PDB 5F2K) (24) and the salicyclic acid carboxyl methyltransferase (CbSAMT, PDB 1M6E) (25). In these enzymes, a His residue is absent in the vicinity of the substrate, but the similar spatial geometry (to CfJHAMT) is retained between the carboxylate moiety of the substrate and the nearby Gln residue and the SAM cofactor. These findings are consistent with the mechanism we proposed for CfJHAMT and likely apply to other JHAMTs, considering the highly conserved nature of the binding pocket for the carboxylate moiety of JHA III.

The role of the N-terminal region of CfJHAMT was further investigated using a specially designed Cleaved-N-6His-Δ9 construct and the Y7F mutant. This region exhibited no observable densities in the absence of substrate binding, hinting at more flexibility. To assess its functional significance, we used the Cleaved-N-6His-Δ9 construct to evaluate its impact on catalysis. Interestingly, this construct demonstrated a dramatic reduction in enzymatic activity, retaining only 11% of the WT activity. Structural analysis of the ternary complex highlighted Tyr7 as a potentially critical residue for catalysis, likely contributing to substrate stabilization through a hydrogen bond between the Tyr7 hydroxyl group and the carboxyl group of the substrate. Supporting this hypothesis, the Y7F mutant exhibited a severe loss of activity, retaining only 9% of enzymatic function, confirming that Tyr7 in the N-terminal region plays a vital role in substrate stabilization. Together, these residues help maintain the spatial alignment and stabilization that support the SN2 methyl transfer reaction, most consistent with a proximity-driven mechanism.

The analysis of the substrate-binding domain reveals a hydrophobic pocket that stabilizes the terpenoid backbone and the epoxide group of JHA III, with Tyr237 playing a critical role in facilitating the interaction with the epoxide. Notably, the stereospecificity observed for JHA III (10R configuration) in the ternary complex aligns with the naturally occurring enantiomer of JH III (26) and favors the interaction between the epoxide and Tyr237. In contrast, the alternative enantiomer would not position the epoxide for equally favorable interaction with Tyr237.

Following these structural observations, biochemical assays were conducted to evaluate the role of Tyr237 in the catalysis of JHA III and FA (a substrate lacking an epoxide group). Consistent with the structurally observed hydrogen bond between Tyr237 and the epoxide group of JHA III, the Y237F mutant exhibited a dramatic loss of activity, retaining only 15% of the catalytic function. Interestingly, for the FA substrate, which lacks an epoxide, catalytic activity was comparable between the wild type and the Y237F mutant, with ∼60% residual activity. This observation indicates that the absence of the epoxide does not hinder substrate binding or enzymatic methylation. However, the removal of Tyr237 significantly disrupts the catalytic conversion of JHA III into JH III, possibly due to impaired substrate stabilization or to the substrate adopting a non-reactive conformation within the active site. This Tyr residue, as highlighted in the protein alignment of functionally tested JHAMTs (27) (Fig. S9), is observed in several Lepidoptera and other holometabolous insects and likely contributes to selectivity for the epoxide group, potentially favoring epoxidized substrates and influencing the order of the last two steps of juvenile hormone biosynthesis, epoxidation and methylation, which can vary among species (1). Other factors, such as the substrate preferences of the epoxidase, are also likely involved (9).

Building on the critical role of Tyr237 in anchoring the epoxide moiety of JHA III, structural comparisons with other JH-bound proteins provided insights into a putative mechanism for juvenile hormone recognition. In the JHA III-bound structure of CfJHAMT, the long hydrophobic cavity is shaped to accommodate the full terpenoid backbone of JHA III, with Tyr237 forming a pivotal hydrogen bond with the epoxide oxygen at the end of this hydrophobic pocket. This interaction is necessary for JHA III binding affinity and is proposed to stabilize the substrate's conformation for catalysis, as evidenced by the drastic reduction in enzymatic activity (retaining only 15%) observed for the Y237F mutant with JHA III. Similarly, in JH-binding proteins (JHBPs), the conserved Tyr residue plays a critical role in hormone binding. The structures of the mosquito JHBP-JH III complex (PDB 5V13) (19) and *B*. *mori* JHBP-JH II complex (PDB 3AOS) were determined using X-ray crystallography, while the *B. mori* JHBP-JH III complex (PDB 2RQF) was determined using NMR spectroscopy (28). The JHBP proteins from these two insects do not share a similar fold, but the binding cavity is always a large and mostly hydrophobic cavity with a Tyr residue interacting with the epoxide (Fig. S10). Namely, Tyr129 in mosquito JHBP and Tyr128 in *B. mori* JHBP form hydrogen bonds to stabilize the epoxide moiety. Similar to our CfJHAMT, these JHBPs rely on this Tyr-mediated hydrogen bonding to stabilize the epoxide moiety, and mutations of this residue to Phe significantly reduce binding affinity. This was demonstrated by isothermal titration calorimetry for mosquito JHBP and radioligand binding assays for *B*. *mori* JHBP (28, 29). Beyond the epoxide group, the carboxylate of JHA III in CfJHAMT forms additional hydrogen bonds, with polar residues further anchoring the substrate. As this Tyr is highly conserved in JHAMT from a large number of insects (in the order Lepidoptera and maybe also in other orders), these findings collectively suggest a unifying mechanism across JHAMT and JH-binding proteins where the hydrophobic cavity provides substrate or ligand binding strength, while the conserved Tyr residue ensures epoxide recognition leading to selectivity and stabilization. In JHAMT, this supports optimal substrate binding and efficient catalysis, whereas in JHBPs, it enables high-affinity hormone binding.

The structural alignment of CfJHAMT and BmJHAMT2 revealed key differences in the substrate-binding pockets, particularly in the JHA III-binding site. Substitutions in BmJHAMT2 result in larger side chains that restrict the pocket size, likely hindering JHA III binding without significant conformational adjustments. These findings highlight a structural basis for the divergence in substrate specificity between CfJHAMT and BmJHAMT2, underscoring the conserved features that align BmJHAMT1 more closely with CfJHAMT.

This supports the hypothesis that BmJHAMT1’s structure, consistent with its known JHA III and FA methylation activity, is likely more similar to CfJHAMT than to BmJHAMT2. Given the above analysis and BmJHAMT2’s very limited methylation activity, the latter enzyme likely contributes minimally to JH biosynthesis under physiological conditions where BmJHAMT1 and BmJHAMT2 are co-expressed. Nevertheless, BmJHAMT2 remains a functional methyltransferase under certain conditions, suggesting that it may accommodate a different class of substrates than JHA III.

In support of this hypothesis, recombinant BmJHAMT2 was shown to form a homodimer in solution, similar to BmJHAMT3, an inactive JHAMT paralogue thought to function as a binding protein for juvenoid acids (30). Structural analysis of superposed BmJHAMT2 and BmJHAMT3 reveals that homodimerization is primarily stabilized by interactions involving Gln202 and Asn259 of BmJHAMT2, which form a hydrogen bond network with their counterparts and the Tyr174 CO backbone. Notably, these critical residues are absent in CfJHAMT and BmJHAMT1, replaced by Glu202 and Lys265 in CfJHAMT and Thr202 and Lys265 in BmJHAMT1 (numbering according to CfJHAMT). The bulkier side chains of these residues cause steric hindrance, likely preventing homodimer formation. This aligns with our observation that CfJHAMT exists as a monomer in solution, while the oligomeric state of BmJHAMT1 remains to be investigated. Overall, these findings point to BmJHAMT1 as being the primary target for further investigation and inhibitor design efforts based on *B. mori*, suggesting that it offers a stronger basis for understanding and targeting JHAMT activity than BmJHAMT2.

It is well established that Lepidoptera, including *C. fumiferana*, synthesizes ethyl-branched JHs—notably JH I and JH II—in addition to JH III (31, 32). *C. fumiferana* incorporates ethyl branches into the juvenile hormone (JH) biosynthetic pathway before the CfJHAMT step, suggesting that CfJHAMT can utilize multiple forms of JH acids, including those with ethyl branches, as substrates. Structural superposition of JHA III with the docked ethyl-branched analogs revealed that the substrate-binding site configuration accommodates all ethyl branches without steric hindrance, suggesting that these structural variations should not impede catalysis (Fig. S11).

The findings presented in this study contribute to a broader understanding of methyltransferase function and suggest that CfJHAMT catalyses the methylation of JHA III with SAM through a proximity and desolvation mechanism. Our results have provided the first prototype of a substrate recognition mechanism by JHAMTs from the highly abundant Lepidoptera order, where methylation is the last step of juvenile hormone maturation. In this process, hydrophobic residues interact with the terpenoid backbone of the substrate, while Tyr237 acts as a critical anchor for the epoxide group. Polar residues at the binding site entrance—Gln14, His115, Trp116, and Tyr7—play pivotal roles in stabilizing and orienting the substrate to facilitate the SN2 methyl transfer reaction. The identification of these key residues and structural domains not only enhances our understanding of JHAMT’s catalytic mechanism but also offers potential targets for modulating its activity. The critical residues identified for JHA III binding and catalysis are highly conserved across JHAMTs, suggesting that other family members likely follow the same recognition pattern. Because juvenile hormone broadly regulates key physiological and developmental processes, insights into JHAMTs enhance our understanding of its maturation. These findings have possible practical implications, particularly for developing pest management strategies aimed at disrupting juvenile hormone biosynthesis in the spruce budworm and other pests.

## Experimental procedures

### Chemicals

Racemic JHA III was purchased from LGC Standards, United Kingdom. JH III and SAH were purchased from Cayman Chemical. FA and MF were purchased from Echelon Biosciences. SAM was purchased from Millipore-Sigma (United States).

### Heterologous enzyme production

The coding region of CfJHAMT was cloned into the expression vector pET28a to generate a fusion protein with an N-terminal polyhistidine tag followed by a thrombin cleavage site. The BL21 Gold (DE3) strain of *E. coli* was then transformed with this construct. Pre-culture was inoculated into a small volume of LB medium supplemented with 50 μg/ml of kanamycin. After overnight incubation at 37 °C with shaking (200 rpm), the pre-culture was used to inoculate 1 L of the same medium incubated under the same conditions until the optical density (600 nm) reached a value between 0.8 and 1.0. Induction under control of the T7 promoter was then initiated by the addition of 0.3 mM isopropyl β-D-1-thiogalactopyranoside. Cultures were incubated on a shaker (150 rpm) at room temperature for 18 h. Bacterial cells were then harvested by centrifugation (6000*g*, 15 min). The cell pellet obtained was resuspended in 25 ml lysis buffer (50 mM Tris-HCl pH 8.0, 400 mM NaCl, 2% glycerol, 5 mM imidazole). The cells were lysed using a sonicator (10 s. ON, 20 s. OFF; 10 min). Cell debris was removed by centrifugation (30,000*g*, 50 min).

For metal affinity chromatography (IMAC), the protein sample was added to cobalt beads (Takara Bio) in a column and incubated with gentle shaking for 30 min at 4°C. Purification was carried out at room temperature with three washing steps following an imidazole gradient. The protein was eluted in elution buffer (50 mM Tris-HCl buffer pH 8.0, 200 mM NaCl, 5% glycerol, 200 mM imidazole). The imidazole was removed using a desalting column EconoPac 10DG (Bio-Rad). The protein was concentrated in a Centricon centrifugal filter (Millipore Sigma, Darmstadt, Germany). The concentration of the protein mixture was estimated using the Bradford method. Protein purity was evaluated by SDS-PAGE. Size-exclusion chromatography was subsequently performed on a Superdex 75 Increase 10/300 Gl column (Cytiva) using an ÄKTA Pure system to estimate the oligomeric state of CfJHAMT (Fig. S4).

### Crystallization

CfJHAMT was concentrated to 15 mg/ml in 20 mM Tris pH 7.5, 5% glycerol, 150 mM NaCl, and 5 mM DTT. Crystallization trials were first carried out using the microbatch-under-oil method (33). Two different crystal forms of CfJHAMT-SAH binary complex were obtained by using the reservoir solutions 0.1 M Magnesium formate, 15%(w/v) PEG 3350 and 0.2 M Calcium chloride, 0.1 M TRIS pH 8.5, 25%(w/v) PEG 4000, respectively. The second CfJHAMT-SAH complex was obtained after cleavage of the N-6His-tag by thrombin. To obtain the CfJHAMT-SAH-JHA III ternary complex, co-crystallization with SAH and JH III was attempted in a microbatch-under-oil with 0.1 M magnesium formate and 15% (w/v) PEG 3350, but the crystals lacked JH III density. These crystals were then used for seeding in hanging drop (34), and the resulting crystals were soaked with 5 mM JHA III for 1 day.

### Data collection, structure determination, and refinement

For data collection at Advanced Photon Source, the crystals were flash-cooled in an N_2_ cold stream using the corresponding reservoir solution supplemented with 15% ethylene glycol as cryoprotectant. The collected data were processed by using XDS package (35). The initial phases of the first CfJHAMT-SAH complex structure were determined by the BALBES molecular replacement pipeline (36) in the CCP4 suite (37). The initial model was further improved by the AutoBuild program (38) in the Phenix package (39). The structure was completed *via* multiple cycles of refinement by Refmac5 (40) and manual model rebuilding in Coot (41). The second CfJHAMT-SAH complex structure and the CfJHAMT-SAH-JHA III ternary complex were determined by MolRep (42) in the CCP4 suite using the first CfJHAMT-SAH structure as the search template. To complete the structure determination, multiple cycles of refinement were carried out using Refmac5 followed by model rebuilding with Coot. The data set for the second crystal form of CfJHAMT-SAH binary complex was initially processed in the space group P21212 by automated pipelines; however, for orthorhombic space groups, refinement stalled with high R-factors (Rfree >40%) that were non-responsive to conventional or twin refinement. Subsequent analysis using Zanuda (43) in CCP4 suite suggested a possible monoclinic lattice (P21), but refinement in P21(unit cell 34.79 × 193.84 × 40.47 Å, β = 90.13°) also resulted in relatively high R-factors as well as a large gap between R and Rfree (R/Rfree = 0.195/0.289 with twin refinement). Finally, refinement was performed in the P1 space group. Twinning was accounted for during refinement in REFMAC5, resulting in a marked improvement in model statistics and electron density maps and yielding the final R/Rfree values of 0.179/0.215. Refinement was carried out using four twin domains with the operators (h, k, l), (−h, k, −l), (h, −k, −l), and (−h, −k, l), with refined twin fractions of 0.29, 0.28, 0.21, and 0.22, respectively. All the models have a reasonably good stereochemistry as analyzed with PROCHECK (44). Data collection and refinement statistics are presented in Table 1. The structures have been deposited with RCSB with the accession codes 9XYO, 9XYQ, and 9XYS.

**Table 1:** X-ray data collection and refinement statistics. (Values in parentheses are for the highest-resolution shell)

Structure	CfJHAMT-SAH Crystal form 1 65 residues disordered out of 273	CfJHAMT-SAH Crystal form 2	CfJHAMT-SAH-JHA III
Space group	P 1 21 1	P1	P 1 21 1
a, b, c (Å) α, β, γ (°)	34.93, 39.22, 104.67, 90, 97.95, 90	40.47, 193.84, 34.79, 90.02, 90.13, 89.98	34.92, 40.74, 96.76, 90, 90.75, 90
wavelength(Å)	1.6039	0.9793	0.9793
resolution (Å)	39.22–2.10 (2.21–2.10)	48.46–2.84 (2.99–2.84)	40.74–1.77 (1.87–1.77)
observed *hkl*	104,888 (14,315)	43,373 (6583)	77,103 (10,688)
unique *hkl*	15,890 (2224)	22,456 (3372)	25,705 (3745)
redundancy	6.6 (6.4)	1.9 (2.0)	3.0 (2.9)
completeness (%)	95.8 (93.5)	90.0 (92.6)	96.0 (96.7)
R_meas_	0.063 (0.913)	0.102 (0.372)	0.049 (0.648)
R_pim_	0.033 (0.488)	0.072 (0.263)	0.027 (0.365)
CC_1/2_	0.999 (0.842)	0.993 (0.874)	0.999 (0.805)
I/(σI)	15.3 (1.9)	4.7 (1.8)	13.4 (2.0)
Wilson B (Å^2^)	40.8	38.1	31.8
R_work_ (# hkl)	0.208 (15142)	0.179 (21313)	0.182 (24440)
R_free_ (# hkl)	0.254 (739)	0.215 (1142)	0.233 (1258)
B-factors (Å^2^) (# atoms) Protein SAH JHA III Water	67.9 (1615) 82.7 (26) / 64.9 (28)	45.3 (8177) 34.2 (104) / 35.6 (18)	37.1 (2207) 31.9 (26) 40.0 (18) 42.1 (99)
Ramachandran			
Most favored (%)	89.1	90.6	92.1
Additional allowed (%)	10.4	9.4	7.4
Generally allowed (%)	0	0	0.4
Disallowed (%)	0.5	0	0
rmsd			
Bonds (Å)	0.0149	0.0098	0.0143
Angles (°)	1.852	1.763	1.855
PDB code	9XYO	9XYQ	9XYS

### Site-directed mutagenesis of CfJHAMT

Site-directed mutagenesis of CfJHAMT was carried out using the QuikChange II Mutagenesis Kit (Agilent technologies) according to the manufacturer’s instructions with the primers listed in Table S1. All mutations were generated and confirmed by Sanger sequencing. The plasmids carrying the mutated genes were transformed into *E. coli* BL21 Gold (DE3) for overexpression. The CfJHAMT mutants were purified using the same protocol as the wild-type CfJHAMT.

### Activity assays of CfJHAMT and mutants

The enzymatic activity of JHAMT was assessed by measuring product formation, based on previously described methods with minor modifications (3, 14). To determine the appropriate reaction time, time-dependent assays were performed, and product formation was quantified by integrating the chromatographic peak area (area under the curve, AUC). These experiments confirmed a linear rate of product formation for both substrates (Fig. S12*A*) and proportionality to enzyme concentration for JH III (Fig. S12*B*), consistent with initial-rate kinetics. Based on these results, all subsequent assays were conducted in a 125 μl reaction mixture containing 50 mM HEPES (pH 8.0), 3 μM enzyme (wild-type or mutant), 400 μM SAM, and 200 μM substrate, incubated at 25 °C for 5 min. Control assays contained no enzyme, and reactions were quenched by the addition of 125 μl of acetonitrile. Denatured proteins were removed by centrifugation. The assays were monitored by HPLC analysis carried out on a reverse-phase column ZORBAX C18 (Agilent technologies, 150 × 4.6 mm, 5 μm) with UV detection at 219 nm under isocratic condition (70% acetonitrile, 30% water for JH III; 80% acetonitrile, 20% water for MF) at a flow rate of 0.5 ml/min for 20 (JH III) or 25 (MF) minutes. Product levels were quantified by AUC. For comparisons between wild-type and mutants, wild-type activity was set to 100%, and mutant activities were expressed relative to this value.

### Molecular Docking simulations

Molecular Docking simulations were conducted using GNINA 1.3.8 (45) with default settings and exhaustiveness set to 64. The autobox_ligand option was defined using the crystallized JHA III ligand to center and size the docking grid. To validate the protocol, a self-docking of JHA III into the CfJHAMT structure was performed. The best-scoring pose reproduced the experimental binding orientation, confirming that the docking parameters accurately depict the known binding mode. Docking poses were ranked by scoring function and filtered by visual inspection, prioritizing conformations that preserved the native orientation of the epoxide–terpenoid core within the catalytic pocket.

## Data availability

The coordinates and structure factors of the CfJHAMT-SAH crystal form 1, CfJHAMT-SAH crystal form 2 and CfJHAMT-SAH JHA III complexes have been deposited in the Protein Data Bank with the accession codes 9XYO, 9XYQ and 9XYS, respectively.

## Supporting information

This article contains supporting information, including additional figures, and table (References (46, 47)).

## Conflict of interest

The authors declare that they have no conflicts of interest with the contents of this article.
