# Supplementary material for: Molecular insights into juvenile hormone maturation by juvenile hormone acid methyltransferase
Source: J Biol Chem. 2026 Apr 17;302(6):111474. doi: 10.1016/j.jbc.2026.111474 (PMC13223930; doi:10.1016/j.jbc.2026.111474)
Supplement: Supporting information [file mmc1.docx]

**Supporting Information**

**Molecular insights into juvenile hormone maturation by juvenile hormone acid methyltransferase**

Marie-Ève Picard^1,2^, Michel Cusson^1,3*^, Rong Shi^1,2*^

^1^ Département de biochimie, de microbiologie et de bio-informatique, Institut de Biologie Intégrative et des Systèmes, Université Laval, Quebec City, QC, G1V 0A6, Canada

^2^ PROTEO, Université du Québec à Montréal, Montreal, QC, H2X 3Y7, Canada

^3^ Natural Resources Canada, Canadian Forest Service, Laurentian Forestry Centre, 1055 du P.E.P.S., C.P. 10380, Station Sainte-Foy, Quebec City, QC, G1V 4C7, Canada

*****Corresponding authors:

RS, [rong.shi@bcm.ulaval.ca](mailto:rong.shi@bcm.ulaval.ca)

MC, [michel.cusson@nrcan-rncan.gc.ca](mailto:michel.cusson@nrcan-rncan.gc.ca) or [michel.cusson297@gmail.com](mailto:miche.cusson297@gmail.com)

Authors’ email addresses:
MÈP, [marie-eve.picard.8@ulaval.ca](mailto:marie-eve.picard.8@ulaval.ca);

**SI Figures**


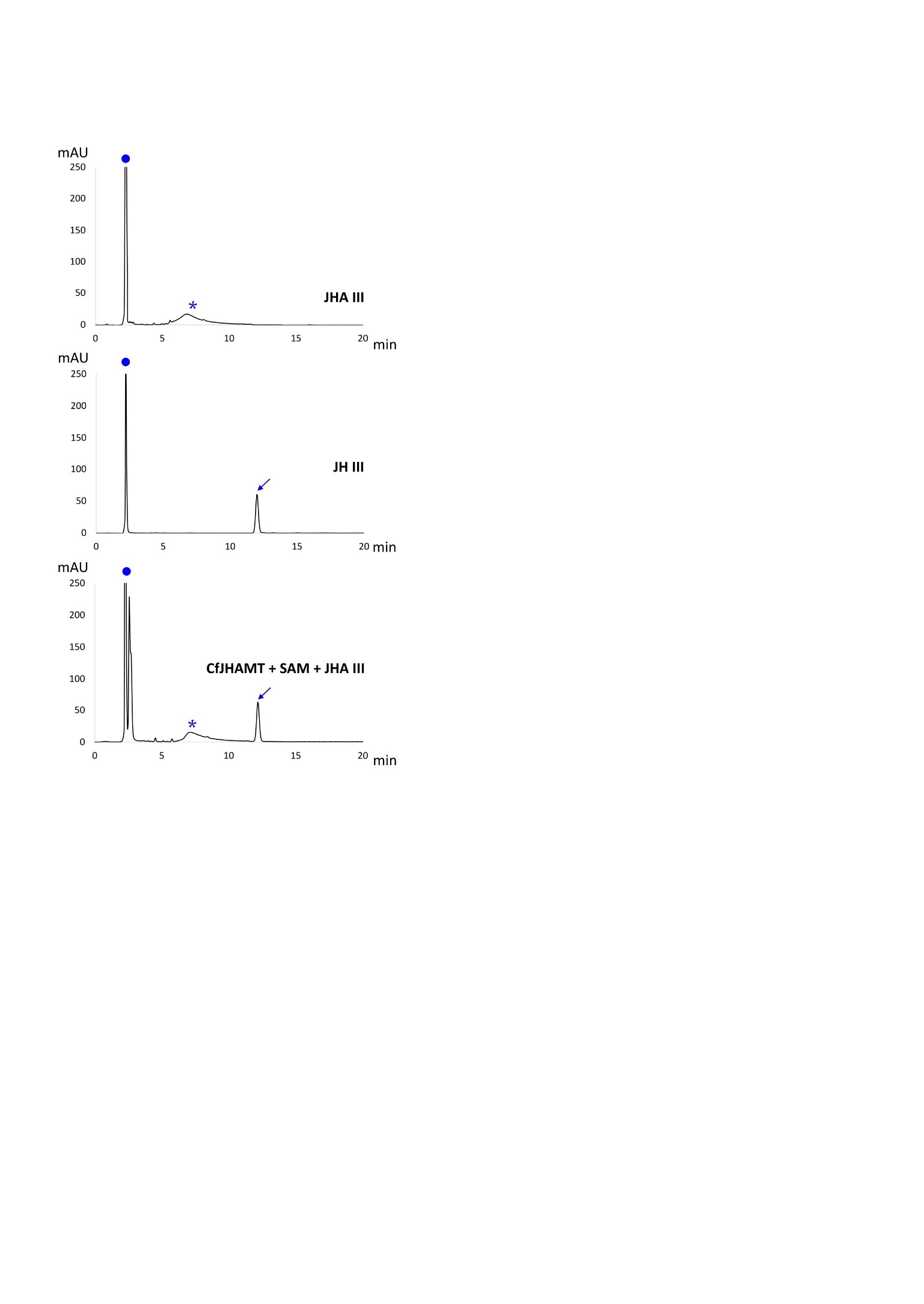


**Figure S1. Activity of recombinant CfJHAMT on JHA III.** Reverse-phase HPLC analysis of JHA III metabolites generated *in vitro*. UV absorbance was monitored at 219 nm. The substrate (JHA III) peak is indicated by an asterisk (*), the product (JH III) peak by an arrow, and unidentified peaks derived from CH₃CN by filled circles (●). Traces show substrate alone (top), product standard (middle), and enzyme reaction (bottom).


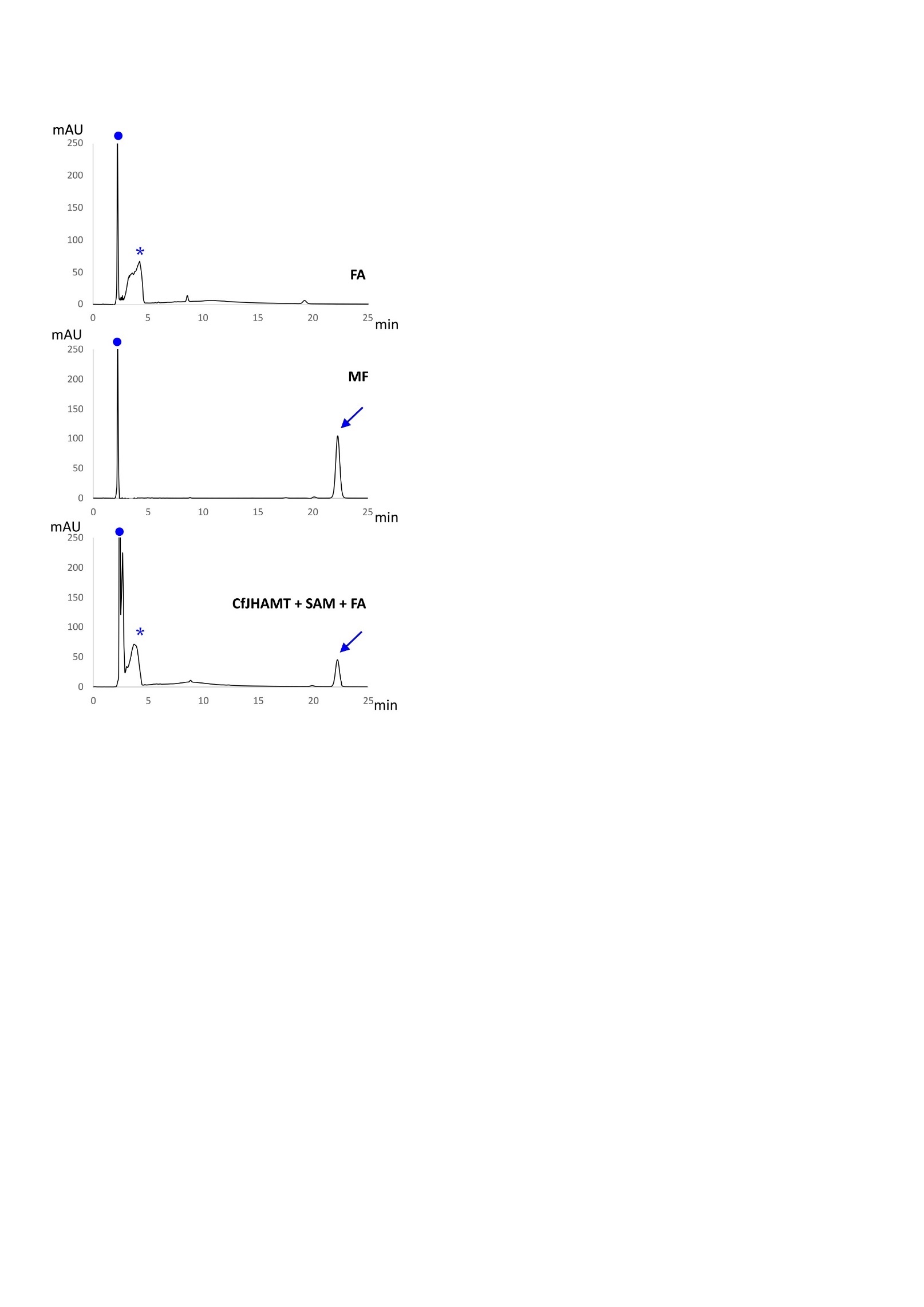


**Figure S2. Activity of recombinant CfJHAMT on FA.** Reverse-phase HPLC analysis of FA metabolites generated in vitro. UV absorbance was monitored at 219 nm. The substrate (FA) peak is indicated by an asterisk (*), the product (MF) peak by an arrow, and unidentified peaks derived from CH₃CN by filled circles (●). Traces show substrate alone (top), product standard (middle), and enzyme reaction (bottom).


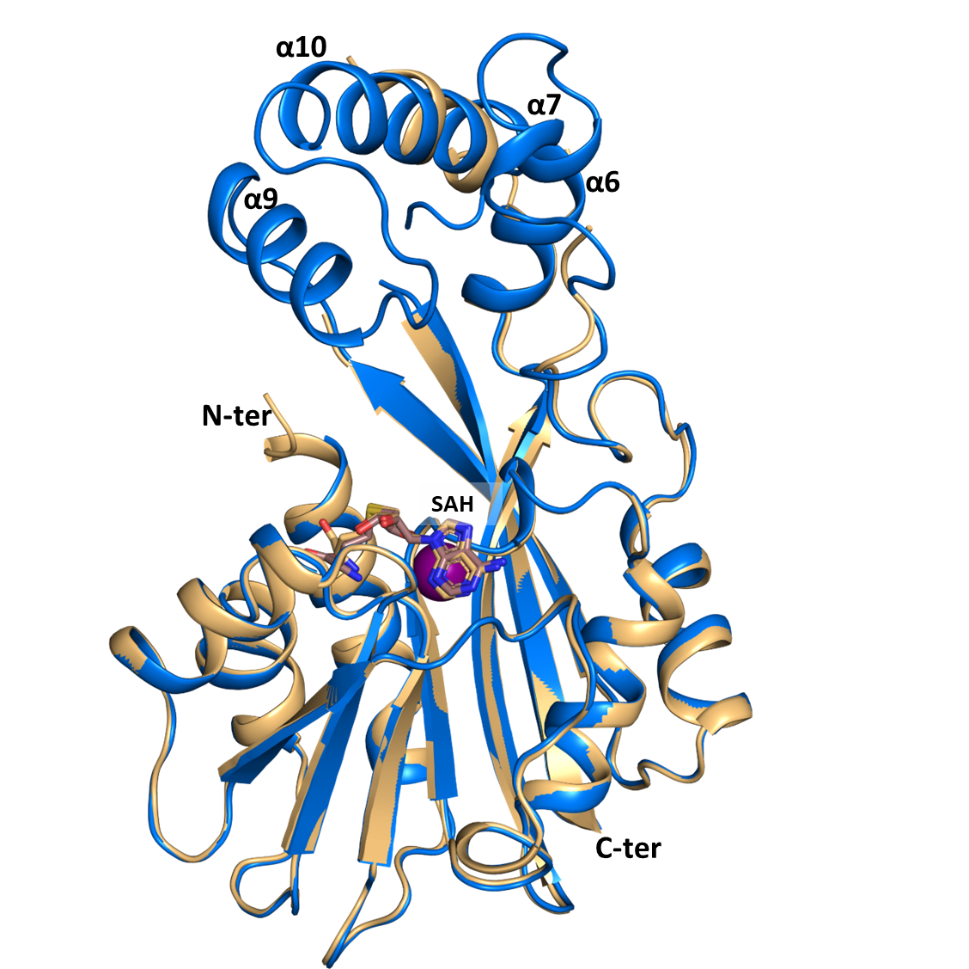


**Figure S3. Comparison of two CfJHAMT–SAH binary structures with varying degrees of order.** The structure colored in wheat has significant missing densities for regions such as helices α9 and α10 compared to the blue CfJHAMT-SAH structure. The former structure contains an iodide ion represented as a purple sphere, incorporated during crystal soaking with sodium iodide initially intended for potential SAD phasing, which was later deemed unnecessary.


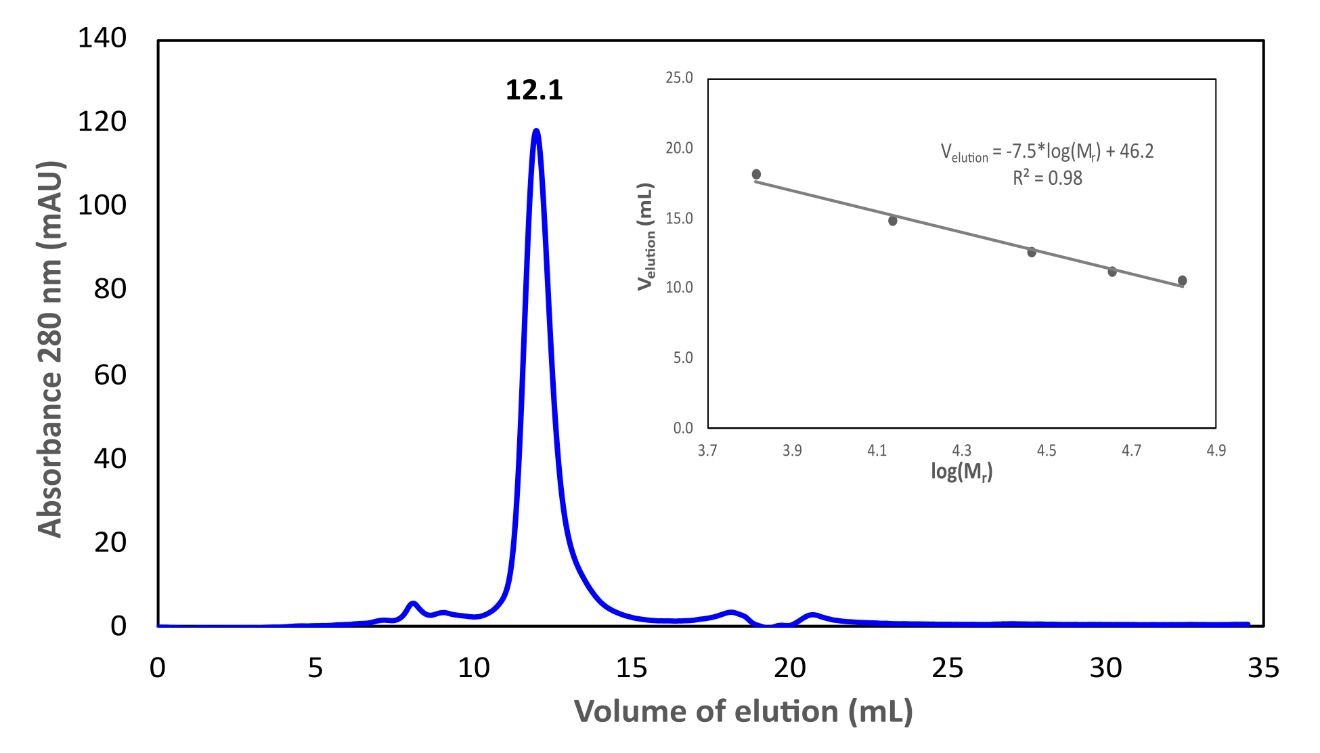


**Figure S4. Size-exclusion chromatography (SEC) profile of recombinant CfJHAMT on a Superdex 75 Increase 10/300 GL column.** The elution profile shows a predominant peak corresponding to CfJHAMT. The inset displays the calibration curve generated using protein molecular weight standards, plotting elution volume versus the logarithm of molecular mass. The elution volume of CfJHAMT (12.1 mL) corresponds to an apparent molecular weight of 35.5 kDa, consistent with a monomeric state.


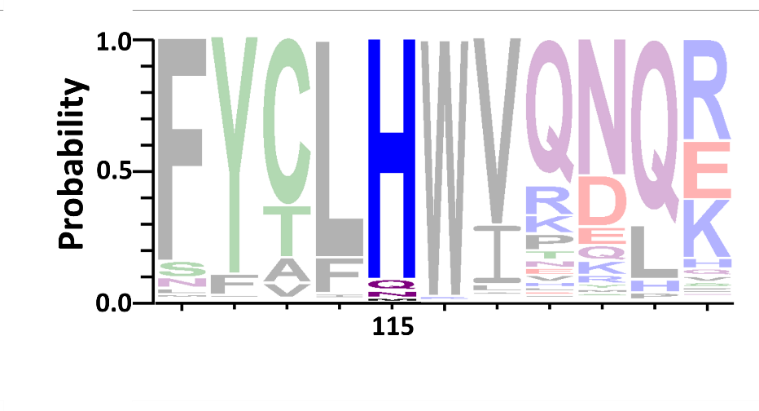


**Figure S5.** **WebLogo (v3.7.9) depiction of residue conservation at the position corresponding to His115 in CfJHAMT, generated from a ClustalW alignment of 500 homologous JHAMT sequences identified by BLASTp (NCBI non-redundant database, default parameters).**


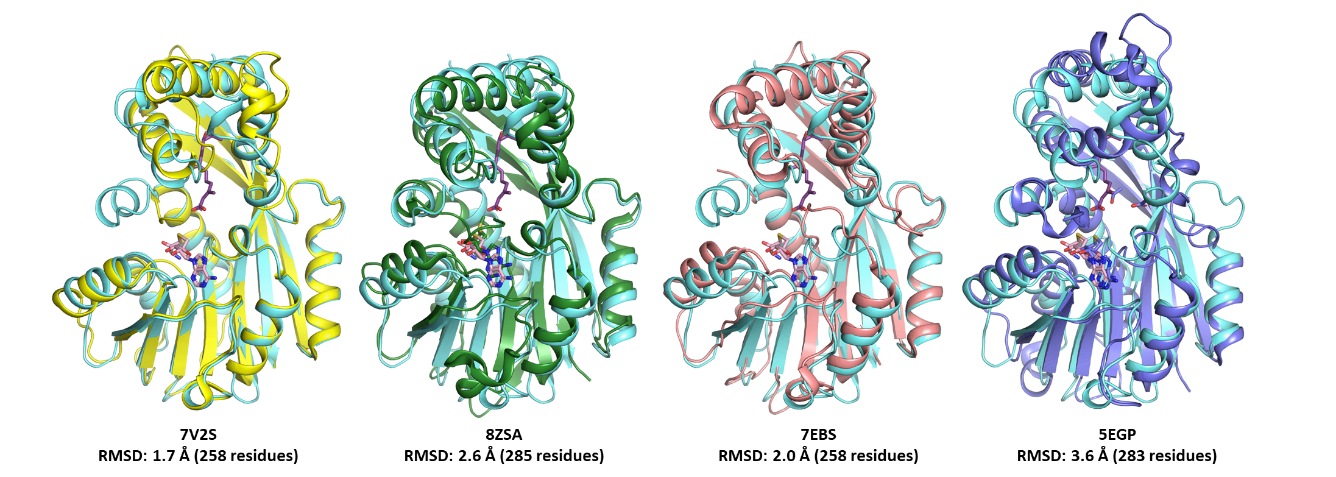


**Figure S6. Structural comparison of CfJHAMT and its closest homologs identified by DALI.**

Using the DALI server, the closest structural homologs of CfJHAMT were identified and compared. CfJHAMT (cyan; this work) is shown alongside BmJHAMT3 (yellow; PDB 7V2S), 4-vinylphenol methyltransferase 2 (4VPMT2; green; PDB 8ZSA), BmJHAMT2_Q15E (pink; PDB 7EBS), and S-methyltransferase TmtA (purple; PDB 5EGP).

**Figure S7. Effect of EDTA on CfJHAMT activity toward JHA III.** Relative activity of mutant protein is expressed as a percentage, calculated by the ratio of JH III production. Assays were conducted using the standard CfJHAMT protocol in 50 mM HEPES buffer (pH 8.0) containing 3 μM enzyme (WT), 400 μM SAM, and 200 μM JHA III substrate, incubated at 25 °C for 5 minutes. Control reactions did not contain any enzyme. Each experiment was performed in triplicate. Mean and standard deviations are shown.


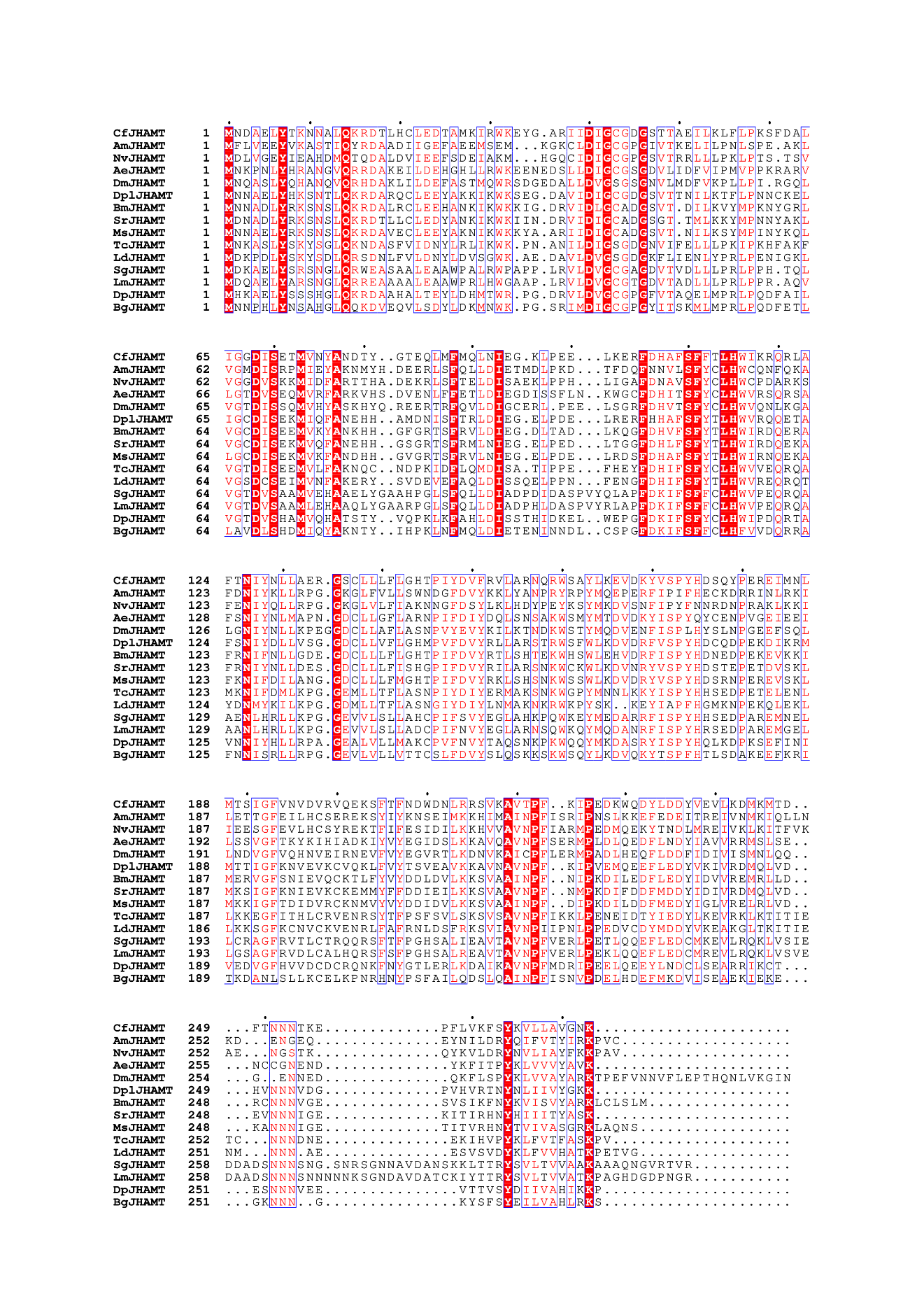


**Figure S8.** Sequence alignment of selected JHAMTs. Species, JHAMT labels, and accession numbers: *Aedes aegypti*, AeJHAMT, EAT42177; *Apis mellifera*, AmJHAMT, XP_001119986; *Blattella germanica*, BgJHAMT, SIW59359; *Bombyx mori,* BmJHAMT, BAC98835; *Danaus plexippus*, DplJHAMT, XP_032515399; *Diploptera punctata*, DpJHAMT, AHZ20738; *Drosophila melanogaster*, DmJHAMT, CG17330 = BAC98836; *Leptinotarsa decemlineata*, LdJHAMT, XP_023011797; *Locusta migratoria,* LmJHAMT, AXM43874; *Manduca sexta*, MsJHAMT, XP_030020606; *Nasonia vitripennis*, NvJHAMT, XP_001604463; *Samia ricini*, SrJHAMT, ABE98256; *Schistocerca gregaria*, ScJHAMT, ADV17350; *Tribolium castaneum*, TcJHAMT, BAG30999.1. The sequence alignment was made with CLUSTALW and rendered using ESPript 3.0 (46).


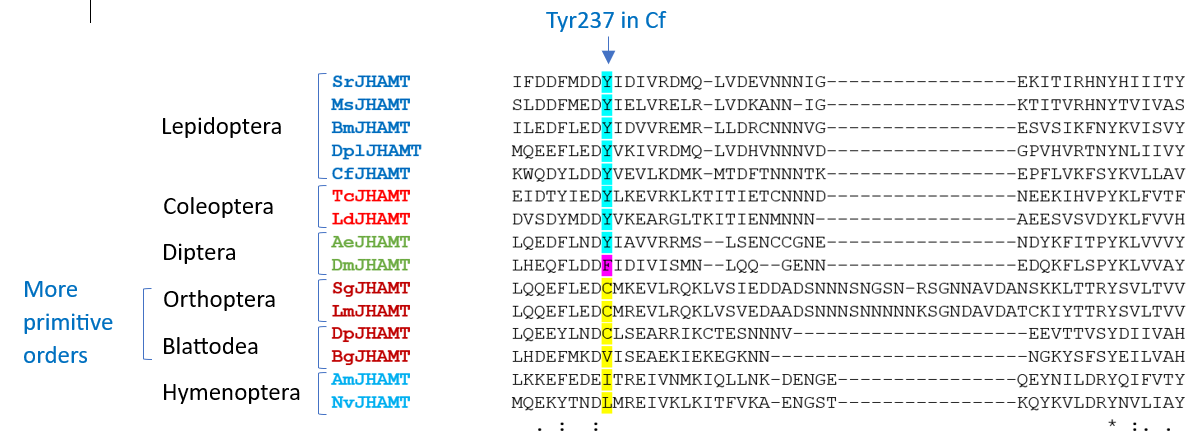


**A**


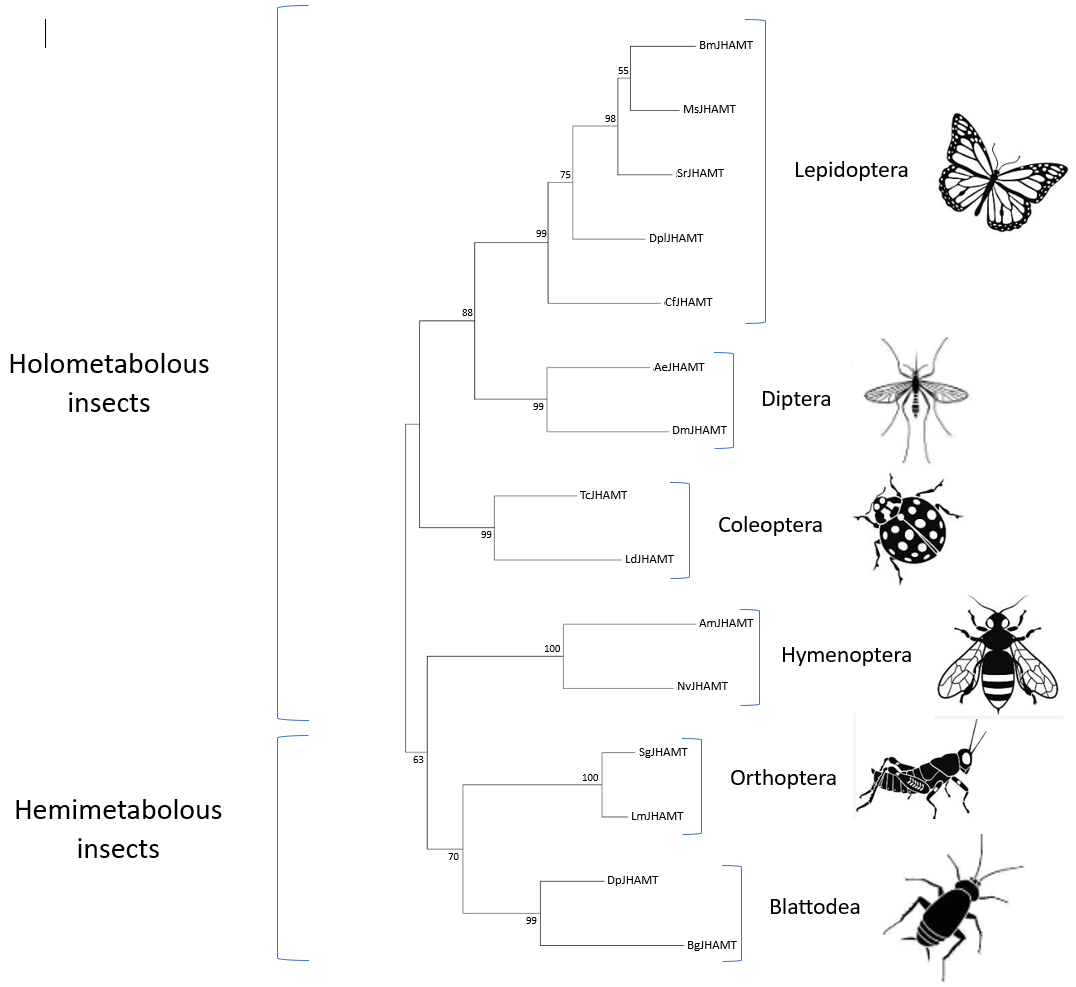


**B**

**Figure S9. Sequence conservation of Tyr237 and phylogenetic analysis of selected insect JHAMTs.** (A) Portion of a JHAMT CLUSTALW alignment comprising 15 species of insects selected from six different orders. The sample alignment highlights the substitutions observed at the position corresponding to CfJHAMT’s Tyr237 in the other JHAMTs. The Tyr residue seems conserved among the Lepidoptera and Coleoptera, partially conserved among the Diptera, but not conserved among the Hymenoptera, Orthoptera and Blattodea. (B) Maximum Likelihood (ML) phylogentic tree inferred using the full JHAMT amino acid sequences of the same 15 species shown in the above alignment. The tree was computed using MEGA X, version 10.1 and the JTT matrix-based model (47)). The percentage of trees in which the associated taxa clustered together is shown next to the branches. This tree highlights the fact that insects from the three orders in which the residue corresponding to CfJHAMT’s Tyr237 is not conserved form a distinct clade relative to the insects of other three orders. Species, JHAMT labels, and accession numbers: *Aedes aegypti*, AeJHAMT, EAT42177; *Apis mellifera*, AmJHAMT, XP_001119986; *Blattella germanica*, BgJHAMT, SIW59359; *Bombyx mori,* BmJHAMT, BAC98835; *Danaus plexippus*, DplJHAMT, XP_032515399; *Diploptera punctata*, DpJHAMT, AHZ20738; *Drosophila melanogaster*, DmJHAMT, CG17330 = BAC98836; *Leptinotarsa decemlineata*, LdJHAMT, XP_023011797; *Locusta migratoria, LmJHAMT*, AXM43874; *Manduca sexta*, MsJHAMT, XP_030020606; *Nasonia vitripennis*, NvJHAMT, XP_001604463; *Samia ricini*, SrJHAMT, ABE98256; *Schistocerca gregaria*, ScJHAMT, ADV17350; *Tribolium castaneum*, TcJHAMT, BAG30999.1.


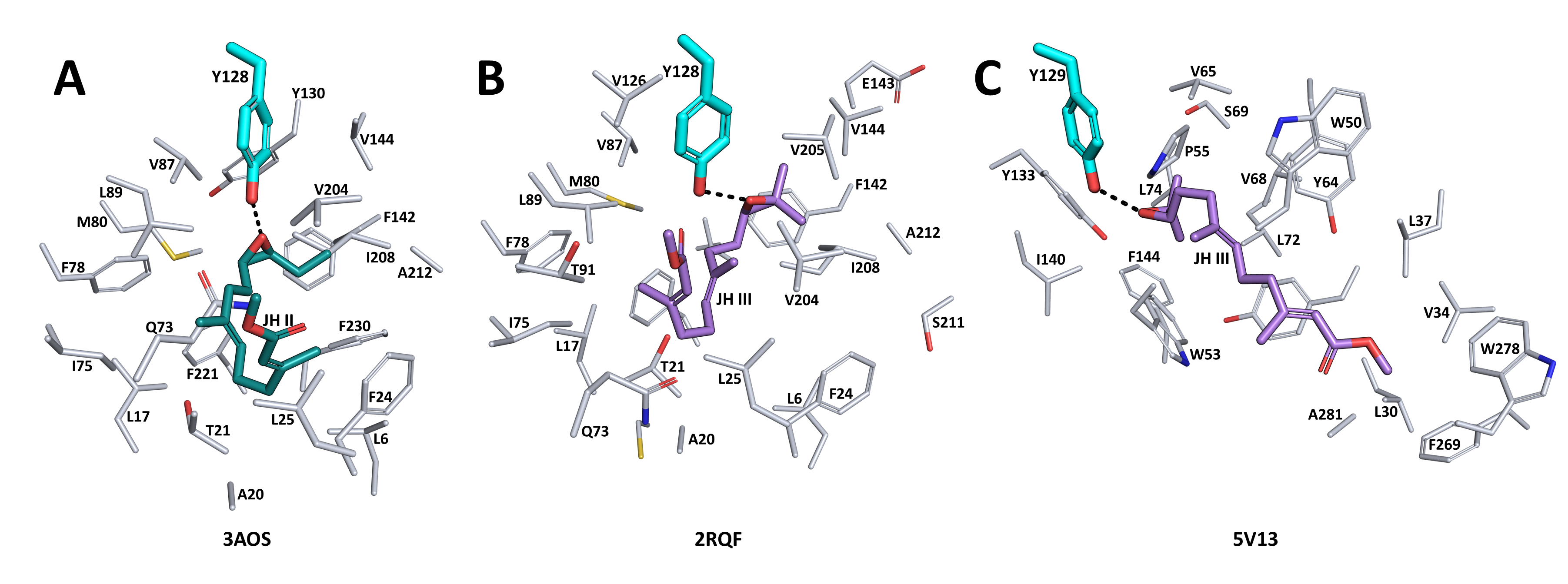


**Figure S10.** Binding cavities of insect JHBPs with conserved Tyr-mediated epoxide stabilization.

(A) *Bombyx mori* JHBP with JH II (PDB: 3AOS). (B) *B. mori* JHBP with JH III (PDB: 2RQF). (C) *Aedes aegypti* JHBP with JH III (PDB: 5V13). The conserved Tyr stabilizing the epoxide (Y128 in 3AOS/2RQF; Y129 in 5V13) is shown in cyan. JH II is in teal, JH III in lavender, and surrounding cavity residues are in gray sticks. Black dashed lines indicate hydrogen bonds between these Tyr and the epoxide moiety.


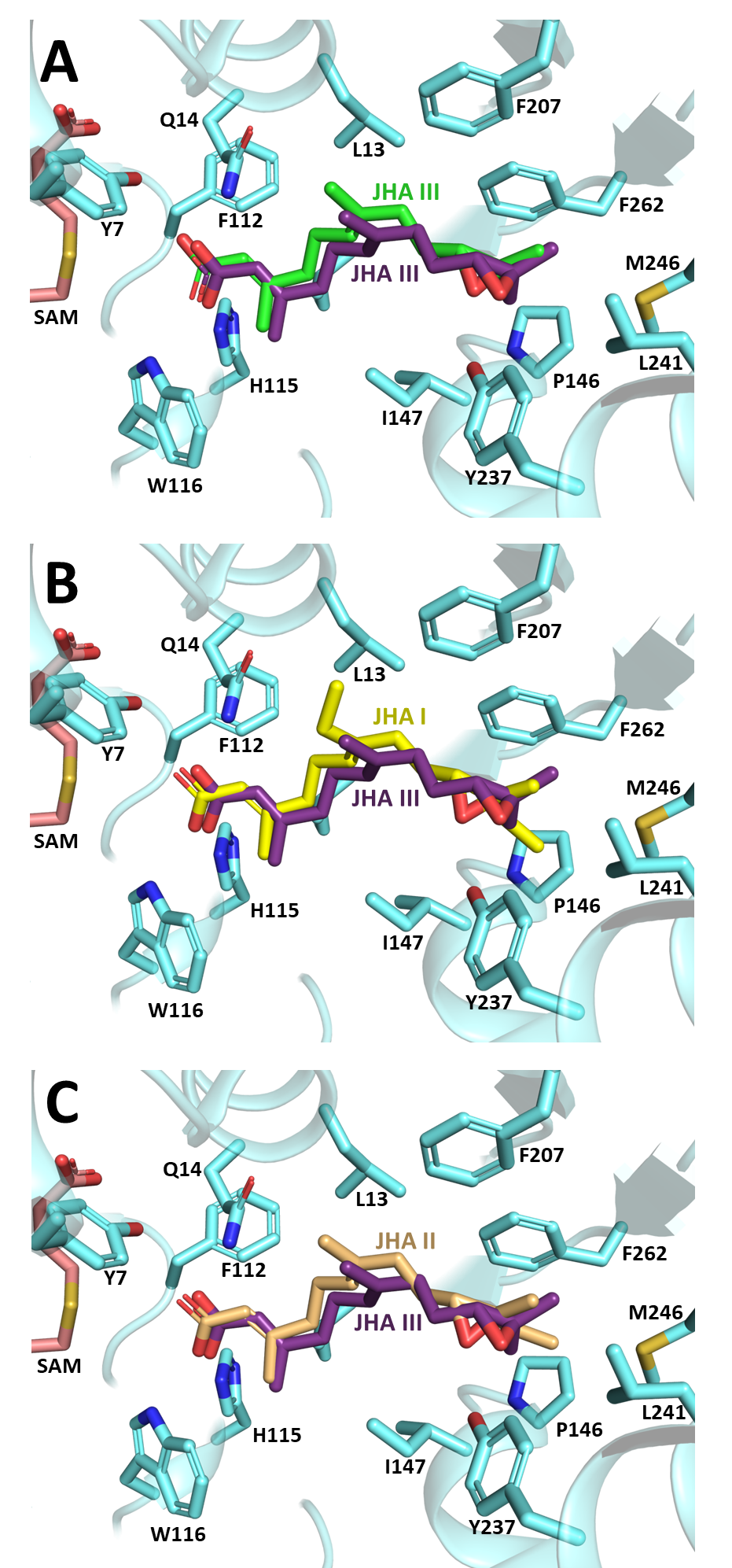


**Figure S11.** Docking of ethyl-branched JHs in the CfJHAMT substrate binding pocket. Docking calculations were performed using GNINA 1.3.8 to assess whether the CfJHAMT substrate binding pocket can accommodate juvenile hormone (JH) variants featuring methyl-to-ethyl substitutions on the terminal aliphatic chain. The acid forms of JH I (JHA I), JH II (JHA II), and JH III (JHA III) were docked. In the validation model (A), the docked JHA III (green sticks) closely overlapped with the crystal-bound JHA III (purple) within the CfJHAMT-SAH-JHA III crystal structure (cyan), confirming the reliability of the docking protocol. (B) Docking with JHA I (yellow sticks) and (C) JHA II (wheat sticks) showed that both ethyl-substituted analogues adopted orientations nearly identical to JHA III, with no steric clashes or displacement within the hydrophobic channel. This consistent spatial alignment indicates that CfJHAMT can accommodate elongation of the aliphatic side chain without inducing an unfavorable binding mode, supporting the idea that the enzyme is structurally permissive toward the natural diversity of JH isoforms.


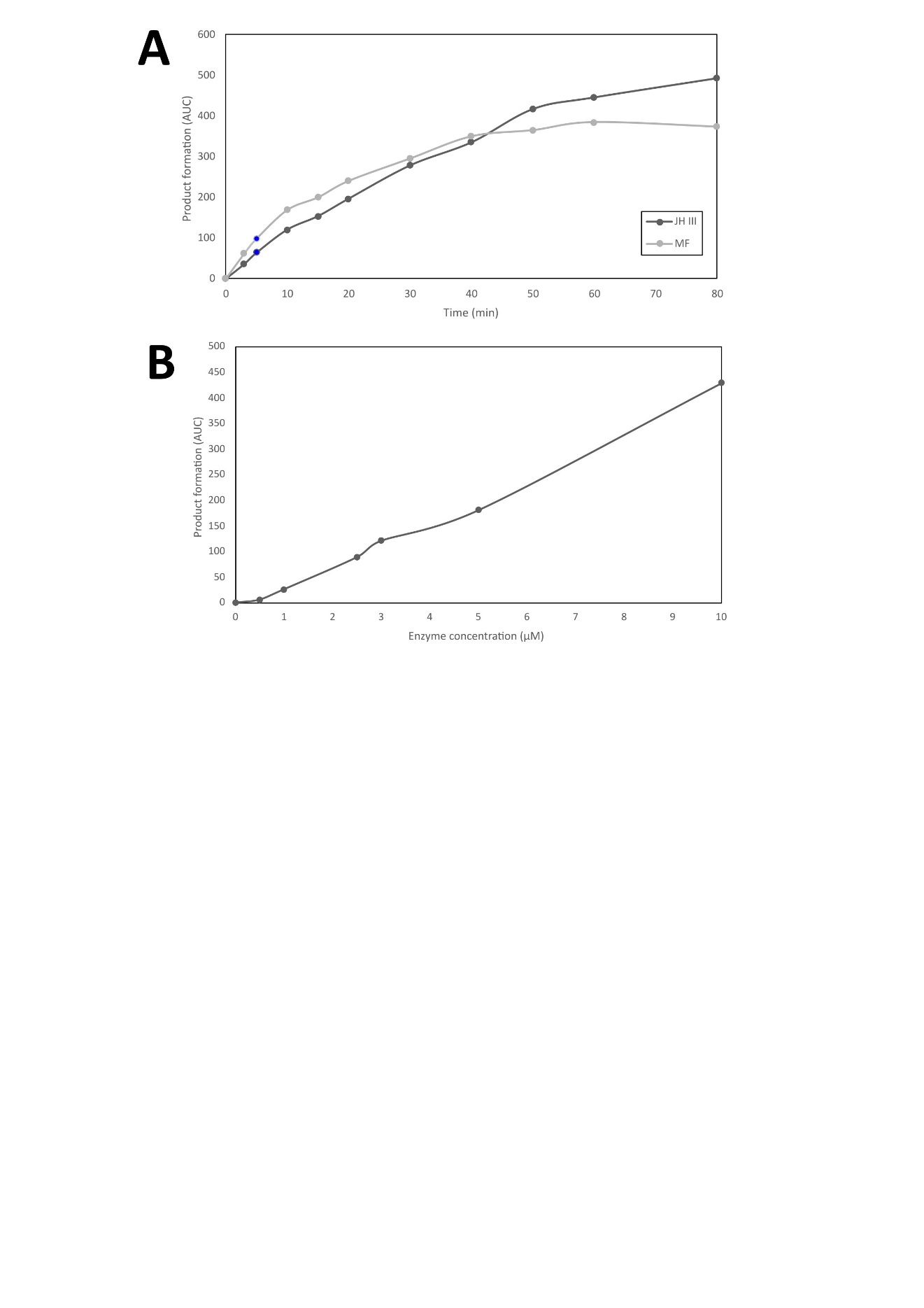


**Figure S12. Validation of initial rate conditions for the CfJHAMT activity assay. (A)** Product (MF, JHA III) formation as a function of time shows linear behavior over the initial time window (≤5–10 min) under the assay conditions. **(B)** Formation of JH III, measured at 5 min, increases linearly with enzyme concentration (0.5–10 µM), confirming proportionality between signal and enzyme concentration. AUC values were measured within the linear range of the assay and are proportional to product formation.

**SI Table**

**Table S1. Oligonucleotides used in this study.**

| Primers | Sequence (5’–3’) |
| --- | --- |
| Δ9_CfMT_F | Gcagcgcattgttatggctgccgcgcg |
| Δ9_CfMT_R | Cgcgcggcagccataacaatgcgctgc |
| Y7F_CfMT_F | catatgaacgatgcggaactgtttaccaaaaacaatgcgctgcag |
| Y7F_CfMT_R | ctgcagcgcattgtttttggtaaacagttccgcatcgttcatatg |
| Q14A_CfMT_F | caaaaacaatgcgctggcgaaacgcgataccctgc |
| Q14A_CfMT_R | gcagggtatcgcgtttcgccagcgcattgtttttg |
| Q14E_CfMT_F | caaaaacaatgcgctggagaaacgcgataccctgc |
| Q14E_CfMT_R | gcagggtatcgcgtttctccagcgcattgtttttg |
| Q14H_CfMT_F | caaaaacaatgcgctgcataaacgcgataccctgc |
| Q14H_CfMT_R | gcagggtatcgcgtttatgcagcgcattgtttttg |
| Q14N_CfMT_F | caaaaacaatgcgctgaacaaacgcgataccctgc |
| Q14N_CfMT_R | gcagggtatcgcgtttgttcagcgcattgtttttg |
| H115N_CfMT_F | cttttttaccctgaactggattaaacgcc |
| H115N_CfMT_R | ggcgtttaatccagttcagggtaaaaaag |
| H115Q_CfMT_F | cttttttaccctgcagtggattaaacgcc |
| H115Q_CfMT_R | ggcgtttaatccactgcagggtaaaaaag |
| V220F_CfMT_F | cgcagcgtgaaagcgtttaccccgtttaaaatt |
| V220F_CfMT_R | aattttaaacggggtaaacgctttcacgctgcg |
| Y237F_CfMT_F | ggattatctggatgattttgtggaagtgctgaaag |
| Y237F_CfMT_R | ctttcagcacttccacaaaatcatccagataatcc |

**References**

46. Gouet, P. (2003) ESPript/ENDscript: extracting and rendering sequence and 3D information from atomic structures of proteins. Nucleic Acids Research. 31, 3320–3323

47. Kumar, S., Stecher, G., Li, M., Knyaz, C., and Tamura, K. (2018) MEGA X: Molecular Evolutionary Genetics Analysis across Computing Platforms. Molecular Biology and Evolution. 35, 1547–1549
